# Enantioselectivity of Chiral Derivatives of Xanthones in Virulence Effects of Resistant Bacteria

**DOI:** 10.3390/ph14111141

**Published:** 2021-11-10

**Authors:** Fernando Durães, Sara Cravo, Joana Freitas-Silva, Nikoletta Szemerédi, Paulo Martins-da-Costa, Eugénia Pinto, Maria Elizabeth Tiritan, Gabriella Spengler, Carla Fernandes, Emília Sousa, Madalena Pinto

**Affiliations:** 1 Laboratory of Organic and Pharmaceutical Chemistry, Department of Chemical Sciences, Faculty of Pharmacy, University of Porto, Rua de Jorge Viterbo Ferreira, 228, 4050-313 Porto, Portugal; fduraes5@gmail.com (F.D.); scravo@ff.up.pt (S.C.); elizabeth.tiritan@iucs.cespu.pt (M.E.T.); madalena@ff.up.pt (M.P.); 2 CIIMAR—Interdisciplinary Centre of Marine and Environmental Research, University of Porto, Novo Edifício do Terminal de Cruzeiros do Porto de Leixões, Avenida General Norton de Matos, S/N, 4450-208 Matosinhos, Portugal; joanafreitasdasilva@gmail.com (J.F.-S.); pmcosta@icbas.up.pt (P.M.-d.-C.); epinto@ff.up.pt (E.P.); 3 ICBAS—Institute of Biomedical Sciences Abel Salazar, Universidade do Porto, Rua de Jorge Viterbo Ferreira, 228, 4050-313 Porto, Portugal; 4 Department of Medical Microbiology, Albert Szent-Györgyi Health Center and Albert Szent-Györgyi Medical School, University of Szeged, Semmelweis utca 6, 6725 Szeged, Hungary; szemeredi.nikoletta@med.u-szeged.hu (N.S.); spengler.gabriella@med.u-szeged.hu (G.S.); 5 Laboratory of Microbiology, Department of Biological Sciences, Faculty of Pharmacy, University of Porto, Rua de Jorge Viterbo Ferreira, 228, 4050-313 Porto, Portugal; 6 CESPU, Institute of Research and Advanced Training in Health Sciences and Technologies (IINFACTS), Rua Central de Gandra, 1317, 4585-116 Gandra, Portugal

**Keywords:** xanthones, chiral, antibacterial, efflux pumps, biofilm, quorum-sensing, enantioselectivity

## Abstract

Antimicrobial peptides are one of the lines of defense produced by several hosts in response to bacterial infections. Inspired by them and recent discoveries of xanthones as bacterial efflux pump inhibitors, chiral amides with a xanthone scaffold were planned to be potential antimicrobial adjuvants. The chiral derivatives of xanthones were obtained by peptide coupling reactions between suitable xanthones with enantiomerically pure building blocks, yielding derivatives with high enantiomeric purity. Among 18 compounds investigated for their antimicrobial activity against reference strains of bacteria and fungi, antibacterial activity for the tested strains was not found. Selected compounds were also evaluated for their potential to inhibit bacterial efflux pumps. Compound **(*R*,*R*)-8** inhibited efflux pumps in the Gram-positive model tested and three compounds, **(*S*,*S*)-8**, **(*R*)-17** and **(*R*,*S*)-18**, displayed the same activity in the Gram-negative strain used. Studies were performed on the inhibition of biofilm formation and quorum-sensing, to which the enantiomeric pair **8** displayed activity for the latter. To gain a better understanding of how the active compounds bind to the efflux pumps, docking studies were performed. Hit compounds were proposed for each activity, and it was shown that enantioselectivity was noticeable and must be considered, as enantiomers displayed differences in activity.

## 1. Introduction

Antimicrobial peptides are proteins produced by several hosts as a defense mechanism against bacteria. They are a response from the innate immune system, and to this day, still present effectiveness against a multiplicity of pathogenic microbes [1,2]. They are composed of amino acids and, therefore, present an amphipathic character, and their cationic charge is able to latch onto the negatively charged bacterial membrane, where they can insert and originate pores, which can ultimately lead to bacterial death [3]. However, several other mechanisms have been brought to light, such as competition with magnesium and calcium ions, bound to lipopolysaccharides in the outer membrane of Gram-negative bacteria, which will lead to membrane destabilization, and, therefore, the entrance of peptides, having a bactericidal effect, either through the leakage of internal organelles or through interaction with internal substances [4].

Since these compounds possess an exposed cationic part and a hydrophobic core, they can establish an initial electrostatic interaction, allowing them to adsorb and aggregate in the negatively charged part of the membrane and also to insert into the membrane due to their hydrophobic moiety [3,4]. These compounds have proven to be effective in both Gram-positive and Gram-negative bacteria [5,6,7,8,9]. In Gram-negative bacteria, they must permeabilize the outer membrane before reaching the cytoplasmic membrane, whereas in Gram-positive they can diffuse through small pores in the peptidoglycan layer [10]. Even though the mechanism of action of these peptides is not fully understood [11], it is known that they can act via membrane disruption and immunomodulation [2]. For instance, in Gram-positive bacteria, antimicrobial peptides can interact with lipoteichoic acid in the cell wall, which by its turn leads to the activation of autolysins, ultimately resulting in bacterial death [12].

Antimicrobial peptides have also been shown to display effective activity against other resistance mechanisms, such as biofilm [13,14] and quorum-sensing, a communication mechanism that modulates the virulence of bacteria [15]. Interestingly, peptides are also chemical signals used for Gram-positive bacteria to communicate via quorum-sensing, which can be used not only for intra-species communication but also with the host [16].

Despite their usefulness, and since these peptides are part of a ubiquitous defense system for eukaryotic organisms, resistance to these compounds has already arisen [17,18], manifesting through multiple mechanisms: electrostatic repulsion of the peptides and modification in the composition of the membrane, repelling the compound and preventing its entrance, respectively [19,20]; peptide degradation or inhibition by proteases and other proteins [21,22]; capsule shedding, a phenomenon triggered by antimicrobial peptides that consist on trapping the peptide within a polysaccharide capsule, thus shielding the bacteria [23]; they can also modulate genes related to resistance [24,25]; and overexpression of efflux pumps, responsible for the increased efflux of antimicrobials from bacteria [26,27].

As such, strategies are being developed to obtain peptide-like molecules with lower manufacturing costs, higher stability, and able to retain activity in the presence of salts. The development of small molecule peptidomimetics has become an attractive field in this sense, and one example is the synthesis of amino acid substituted xanthones. In fact, the substitution of the hydrophobic xanthone core with cationic peptide moieties has shown promising results for the development of new antibacterial agents [28,29]. Our group has already described xanthones as antimicrobials in susceptible and resistant strains [30,31], and very recently as inhibitors of antimicrobial resistance mechanisms [32]. Moreover, enantioselectivity studies associated with the biological activity of chiral derivatives of xanthones and thioxanthones were also performed by our group [33,34,35,36,37]. Actually, for some chiral derivatives, enantioselectivity was found in tumor cell growth inhibition [33,34,37], cyclooxygenases inhibition [36], and P-glycoprotein (P-gp) induction [35].

Regarding antimicrobial activity, it is important to highlight that the configuration of the stereogenic centers is often ignored, and only a few examples describe the activity for both enantiomers [38,39]. Nevertheless, differences in the antimicrobial activity between enantiomers or epimers have been observed. One example concerns the naturally occurring epimers of scortechinone A and L, being scortechinone L more active against bacteria [40].

Herein, new molecular modifications to the xanthone scaffold were planned in order to synthesize chiral peptide-like derivatives and investigate their antimicrobial activity, as well as mechanisms of antibacterial resistance, specifically inhibition of efflux pumps, biofilm formation, and quorum-sensing. The synergy of these compounds with a β-lactam antibiotic in a resistant strain of a clinically relevant bacterial species was also studied. Docking studies were performed to gain a better understanding of how these compounds could interact with relevant bacterial efflux pumps. For each chiral peptide-like derivative, both enantiomers were synthetized, in high enantiomeric purity, to explore enantioselectivity in all assays. The results obtained show the potential of these compounds acting in different mechanisms, suggesting these chiral peptide-like xanthones could be useful in tackling antimicrobial resistance.

## 2. Results and Discussion

### 2.1. Chemistry

**Scheme 1:**
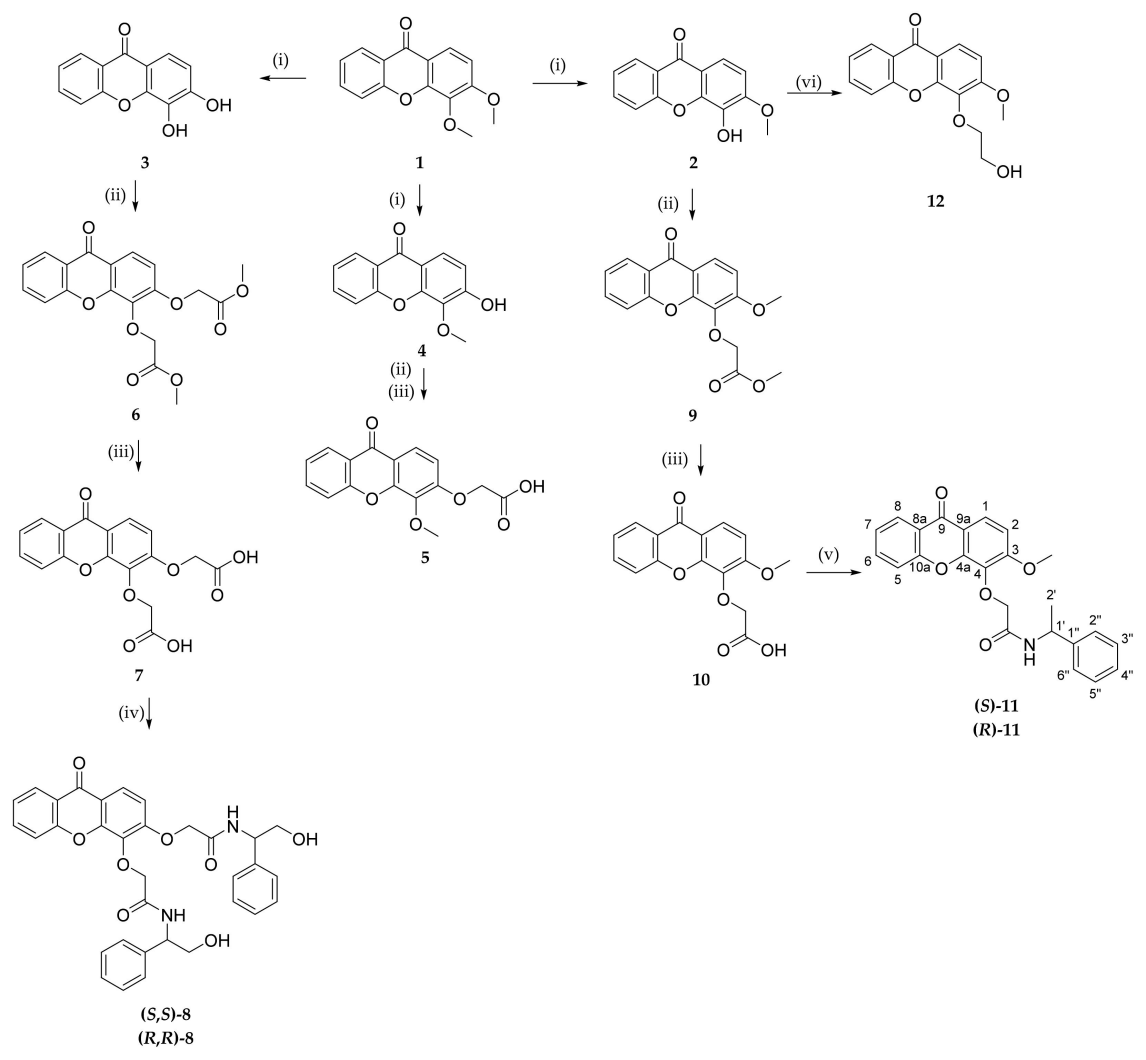
Synthetic pathways for obtaining 3,4-substituted chiral derivatives of xanthones used in this study. (i) AlCl_3_, anhydrous toluene, 70 °C, 40 min; (ii) BrCH_2_COOCH_3_, K_2_CO_3_, anhydrous acetone, 4–24 h; (iii) NaOH 5M, CH_2_Cl_2_:CH_3_OH (1:1 *v/v*), rt, 5–24 h; (iv) (*R*)-(−) or (*S*)-(+)-2-phenylglycinol, TBTU, anhydrous THF, TEA, rt, 5 h; (v) (*R*)-(+) or (*S*)-(−)-(α)-methylbenzylamine, TBTU, TEA, anhydrous THF, 2 h; (vi) BrCH_2_CH_2_OH, NaH, anhydrous acetone, reflux, 14 h.

**Scheme 2:**
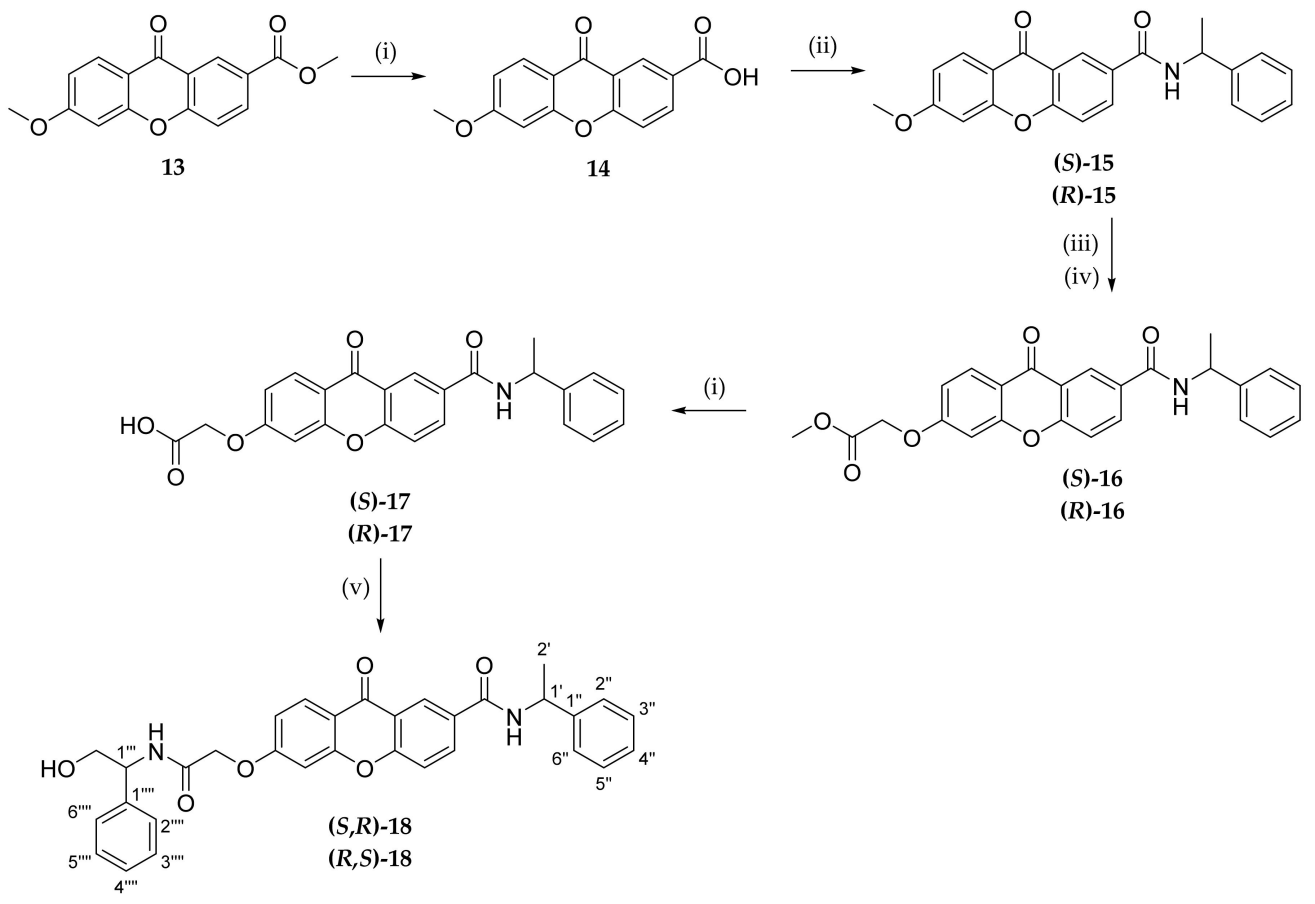
Synthetic pathways for obtaining 2,6-substituted chiral derivatives of xanthones used in this study. (i) NaOH 5M, CH_2_Cl_2_:CH_3_OH (1:1 *v/v*), rt, 5–22 h; (ii) (*R*)-(+) or (*S*)-(−)-(α)-methylbenzylamine, TBTU, anhydrous THF, TEA, rt, 1 h; (iii) Et_2_NCH_2_SH.HCl, NaO*t*Bu, anhydrous DMF, reflux, N_2_, 4 h (iv) BrCH_2_COOCH_3_, K_2_CO_3_, anhydrous acetone, 3 h; (v) (*S*)-(−) or (*R*)-(+)-2-phenylglycinol, TBTU, anhydrous THF, TEA, rt, 3 h.

A library of 18 xanthone derivatives with different substitution patterns in one ring (3,4-substituted) or both rings (2,6-substituted) of the benzopyrano scaffold was synthesized, starting from hydroxy-(Scheme 1) or carboxy-xanthone (Scheme 2) building blocks.

The synthesis of the methoxylated xanthone **1** has also been previously described, through a benzophenone intermediate, which produced the main product 3-methoxy-4-hydroxy-9*H*-xanthen-9-one (**2**), but also the secondary products 3,4-dihydroxy-9*H*-xanthen-9-one (**3**) and 3-hydroxy-4-methoxy-9*H*-xanthen-9-one (**4**), which were used for the synthesis of derivatives [41]. Amines and amino alcohols were chosen as chiral building blocks, to demonstrate the efficiency and applicability of this methodology, resulting in a peptide bond. Activation of a carboxyl group for the formation of peptide bonds can be achieved by in situ activation using coupling reagents [42].

First, a direct coupling reaction to the phenol of compound **2** with a chiral building block using (1-cyano-2-ethoxy-2-oxoethylidenaminooxy) dimethylamino-morpholino-carbenium hexafluorophosphate (COMU) was attempted, although many secondary products were obtained, demonstrating to be an inefficient process. The same reaction was also attempted with 2-(1*H*-benzotriazole-1-yl)-1,1,3,3-tetramethyluronium tetrafluoroborate (TBTU), but the results were also not satisfactory. In order to accomplish this coupling with chiral building blocks, a short spacer was introduced through a Williamson ether synthesis with methyl 2-bromoacetate affording compound **9**. After an alkaline hydrolysis of the ester (**10**), coupling reactions with suitable chiral building blocks were performed, using TBTU, affording the desired products (both enantiomers of **11**).

Coupling reactions were carried out in anhydrous conditions in order to prevent hydrolytical degradation and the formation of secondary products [43]. These reactions occurred at room temperature with 2 equivalents of triethylamine (TEA) and a slight equivalent excess of coupling reagent and chiral building block (1.1 equivalent). These reactions were carried out with both enantiomers of enantiomerically pure commercial building blocks with no tendency towards racemization or enantiomeric interconversion. The building blocks (*R*)-(−) and (*S*)-(+)-2-phenylglycinol, and (*R*)-(+) and (*S*)-(−)-(α)-methylbenzylamine have a primary amine as a reactive group for amide synthesis. Purification processes involved chemical extractions and filtration steps, flash chromatography, and crystallization.

The synthetic pathways used for the syntheses of compounds **6**–**12** are displayed in Scheme 1.

The synthesis of xanthones **13**, **14,** and the enantiomeric pair **15** was previously described [37,44]. Then, to couple an additional chiral moiety in the C6 position, a demethylation step took place, followed by a Williamson ether synthesis with methyl bromoacetate (**16**). In order to perform the coupling with the chiral building blocks, the ester was hydrolyzed to a carboxylic acid (**17**), thus allowing the coupling using TBTU to afford the final compounds **18**. Scheme 2 shows the syntheses leading to 2,6-substituted chiral derivatives of xanthones **16**–**18**.

**Figure 1:**
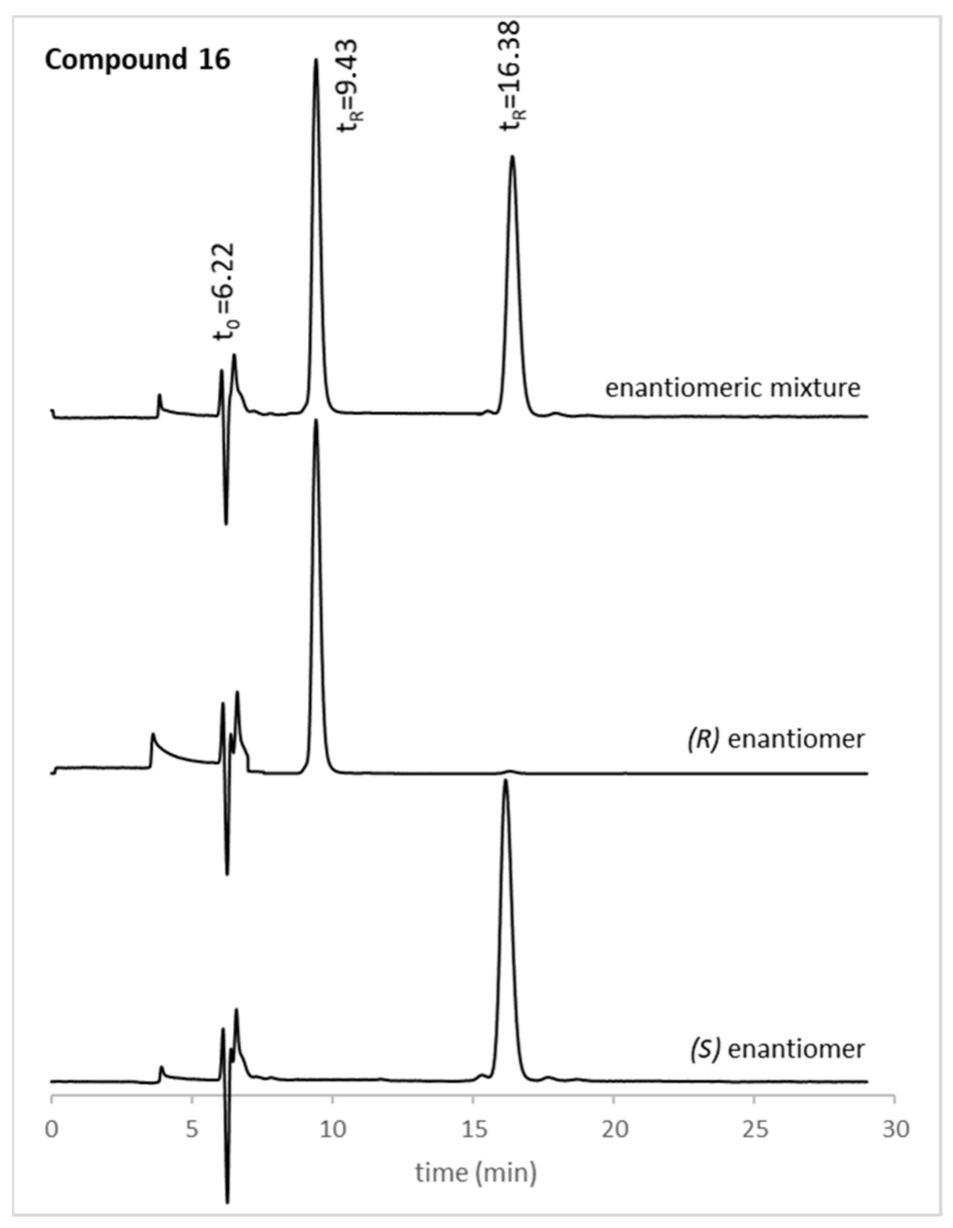
Chromatograms for the enantioseparation of the enantiomeric mixture **16**, at optimized chromatographic conditions.

Xanthones **1**–**4** have already been studied for their potential as inhibitors of bacterial resistance mechanisms [32], therefore, for this study, derivatives **5**–**12**, **17**, and **18** were tested. The structures of the compounds used herein were elucidated by nuclear magnetic resonance (NMR), Fourier-transform infrared spectroscopy (FTIR), and high-resolution mass spectrometry (HRMS). The optical rotation was also determined, and the enantiomeric purity was evaluated by chiral liquid chromatography (cLC). The (*S*,*S*)-Whelk-O1^®^ column and a mixture of CH_3_CN/CH_3_OH (50:50 *v/v*) (compounds **11**, **16**, and **18**) or 2-propanol/*n*-hexane/acetic acid (80:20:0.1 *v/v*) (compound **17**) were chosen as mobile phase, based on the experience of our group [45,46]. Generally, the enantiomeric ratio (e.r.) values for all xanthone derivatives were higher than 99%, as exemplified in Figure 1 for the enantiomeric pair **16**. The optical rotation and e.r. values of the new chiral xanthones can be found in Section 3.1.

### 2.2. Docking Studies

The potential of the 10 synthesized chiral xanthones (both enantiomers of **8**, **11**, and **16**–**18**), as well as 6 precursors (**5**–**7**, **9**, **10**, and **12**), as bacterial efflux pump inhibitors, was investigated through docking studies. These were aimed at comparing the results obtained with compounds previously described as bacterial efflux pump inhibitors. The models used were the three components of the AcrAB-TolC efflux pump, an efflux system that belongs to the resistance-nodulation-division (RND) family, which is relevant in Gram-negative bacteria, and are deposited in the Protein Data Bank (AcrA: 2F1M; AcrB: 4DX5; TolC: 1EK9) and the NorA efflux pump, which belongs to the major facilitator superfamily (MFS) and is predominantly found in Gram-positive bacteria. NorA does not have crystal structure deposited in the Protein Data Bank, therefore, a homology model was built and used for the docking studies. The sites studied were sites described as important in the literature. For the periplasmic adaptor AcrA, the helical hairpin (HH) and the lipoyl domain (LD) were chosen [47]. The transmembrane pump AcrB was described to have a substrate-binding site (SBS) and a hydrophobic trap (HT), whose residues were picked for these studies [48], and the outer membrane channel TolC, was studied in the site comprised by the lysine residues that interact with the 3,3′-dithiobis(sulfosuccinimidyl propionate) bifunctional crosslinker [47]. The homology model of NorA was built as previously described [49], and thus docking studies were performed in the sites used in the same study, which were the binding core region (BCR) and the cytoplasmic side (CS) [49]. Compounds with reported activity in the efflux systems of Gram-positive and Gram-negative were used as positive controls, such as reserpine, phenyl-arginyl-β-naphthylamide (PAβN), D13-9001, doxorubicin, MBX-3132, and minocycline [50]. The results of the docking studies are shown in Table 1.

From the analysis of Table 1, some conclusions can be inferred. First, it can be noted that, as a general premise, the compounds were predicted to act similarly to the positive controls tested herein. Within the AcrAB-TolC efflux system, compounds present the most favorable predicted affinities towards the AcrB portion, specifically the SBS, and the least favorable for AcrA, similarly to the previously described for simple oxygenated xanthones [32].

The analysis of the enantiomeric pairs also led to interesting remarks, as their predicted affinities were different between the two enantioderivatives. For instance, the *S*-enantiomer of compound **8** has a better-predicted affinity for the AcrA portion and the NorA efflux pump, while the *R*-enantiomer is predicted to have a better fit in the AcrB portion, and both enantiomers appear to interact with TolC in the same manner, as their docking scores were the same. Compound **11** shows a notable difference concerning the HT of AcrB, with the *R*-enantiomer presenting a much favorable docking score for this site. The other compounds present values similar to what was described for compound **8**, which led to hypothesize that the substituents linked to the chiral center may be involved in the interactions of these compounds with the referred targets.

### 2.3. Antimicrobial Activity and Synergy with Antimicrobials

Compounds **5–12** and **16–18** were evaluated for their antimicrobial activity against three Gram-negative bacterial strains, *Escherichia coli* ATCC 25922, *Pseudomonas aeruginosa* ATCC 27853 and *Salmonella enterica* serovar Typhimurium SL1344 (SE03), with the *acrA* gene deleted, and three Gram-positive strains, *Staphylococcus aureus* ATCC 29213, *Enterococcus faecalis* ATCC 29212 and the methicillin and ofloxacin-resistant clinical isolate *S. aureus* 272123. For the synergy assay, the extended-spectrum β-lactamase (ESBL)-producing *E. coli* SA/2 [51] was used, and the antibiotic cefotaxime (CTX) was chosen for observation of a decrease in the minimum inhibitory concentration. The antifungal activity of these compounds was also investigated for the yeast *Candida albicans* ATCC 10231, the filamentous fungi *Aspergillus fumigatus* ATCC 204305, and the dermatophyte *Trichophyton rubrum* FF5. The results show that no compound presented observable inhibition of bacterial growth, being their MIC values over 100 µM.

Concerning the synergy assay, compounds **6** and **(*S*)-11** can decrease the MIC of CTX, a β-lactam to which *E. coli* SA/2 is resistant. The observed MIC of CTX in this bacterial strain was 562 µM (256 µg/mL), and it was decreased to 141 µM (64 µg/mL) in the presence of a non-toxic concentration of compounds **6** and **(*S*)-11**. This suggests that these compounds may be able to interact with ESBL and may pose a viable strategy to adopt in combination with antimicrobials. The fact that compounds **6** and **(*S*)-11** do not present antibacterial activity, make them less prone to the appearance of resistances. Once again enantioselectivity was found.

All the tested compounds displayed MIC above 128 µg/mL for the fungal strains tested. The results obtained in these assays are summarized in Table S1 (Supplementary Data).

### 2.4. Efflux Pump Inhibition

In order to study the potential of the compounds to act as efflux pump inhibitors, 10 chiral derivatives and their precursors were studied for their ability to modulate the accumulation of ethidium bromide (EB), a known efflux pump substrate that can increase fluorescence when bound to DNA [52]. The bacteria chosen for this assay were the Gram-positive *S. aureus* 272123, a clinical strain resistant to methicillin and ofloxacin, and the Gram-negative *Salmonella* Typhimurium SE03, with the *acrA* gene deleted, as this was predicted to be the portion of the efflux system to which the compounds would present the least affinity. These models were chosen to compare the activity of the derivatives herein present with the previously described activity of structurally related xanthones and thioxanthones [32,53].

The compounds were tested at 50 µM, as none presented antibacterial activity for the tested strains. Reserpine and carbonyl cyanide 3-chlorophenylhydrazone (CCCP) were chosen as positive controls for *S. aureus* 272123 and SE03, respectively, at the sub-MIC concentration of 25 µM. The results were expressed as the relative fluorescence index (RFI), which was calculated based on the mean of relative fluorescence units and can be seen in Table 2.

**Figure 2:**
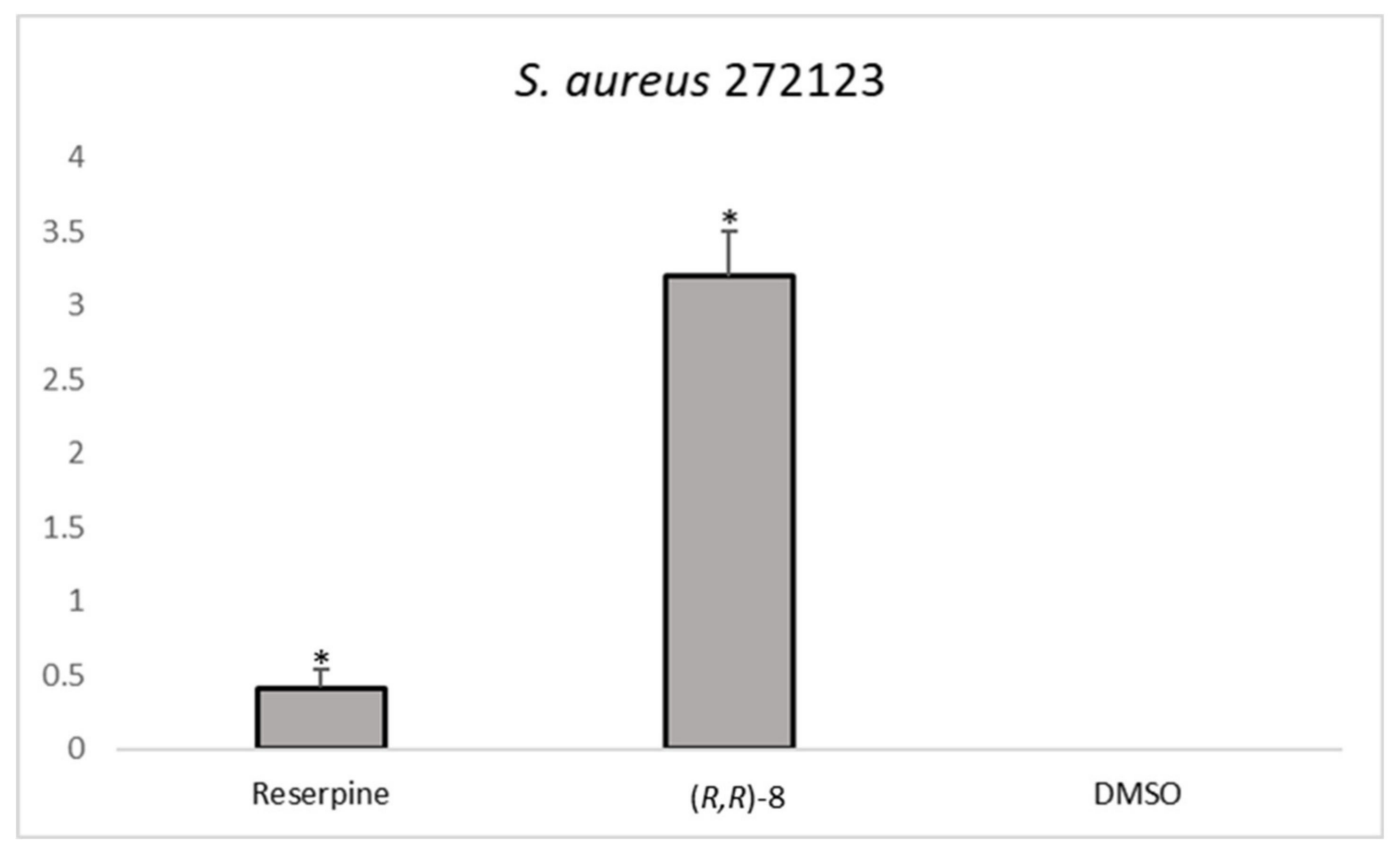

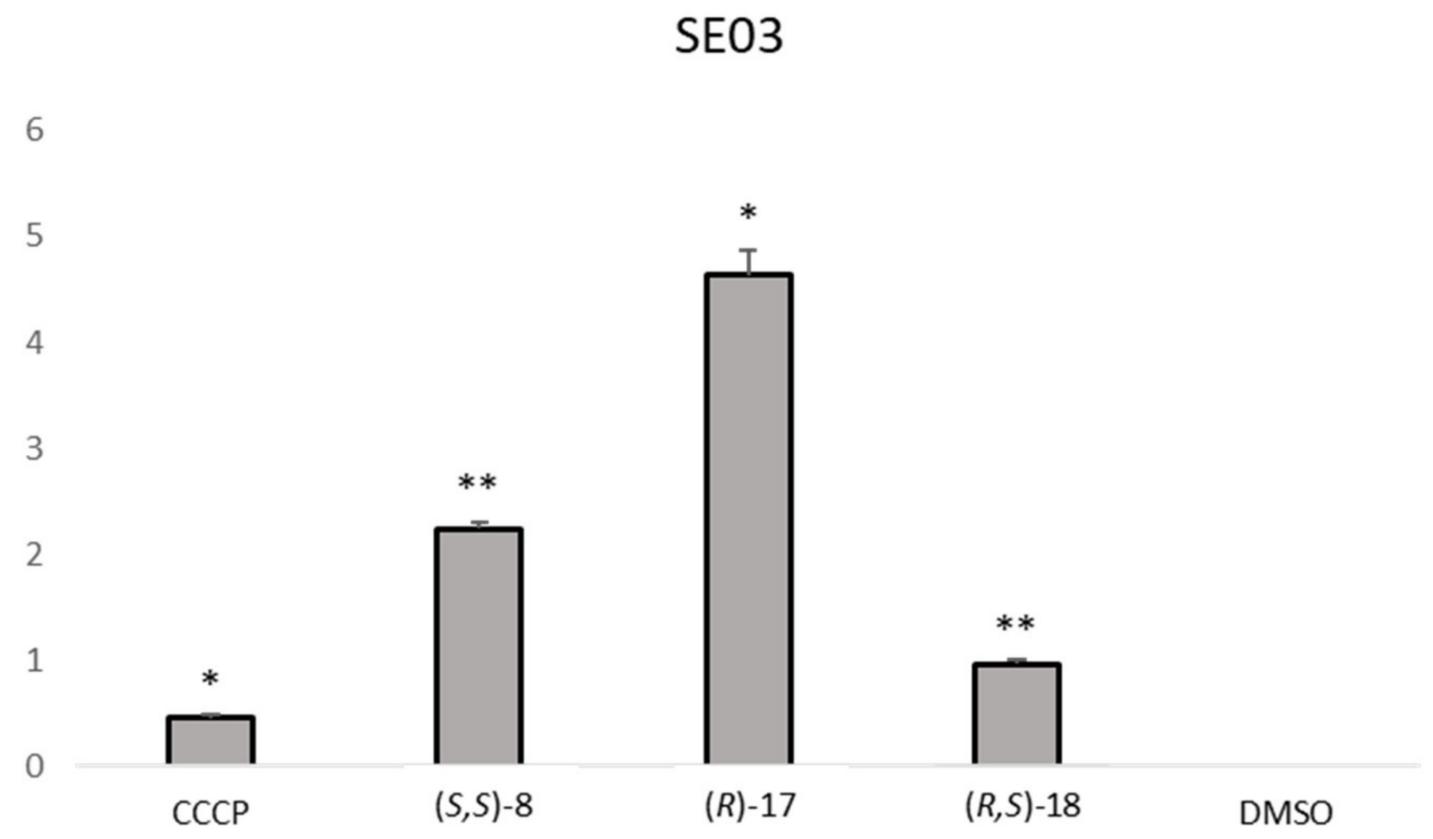
Comparison of the RFI of different compounds to those of the positive controls [*S. aureus* 272123 (**top**); SE03 (**bottom**)]. Results are presented as mean ± SD. Statistical comparisons were performed using the *t*-test [* *p* < 0.05; ** *p* < 0.01 vs. control (DMSO 1% *v/v*)].

The analysis of the RFI showed that one compound, **(*R*,*R*)-8**, was effective in increasing the relative fluorescence in *S. aureus* 272123 in a higher level than reserpine. On the other hand, three compounds were effective in the display of the same effect in SE03, **(*S*,*S*)-8**, **(*R*)-17** and **(*R*,*S*)-18**. This event could be attributed to the inhibition of EB efflux, but also to the fluorescence of the compounds. As such, the RFI of the compound alone and in combination with EB was measured over the same period in order to observe if the compounds displayed an erratic pattern of fluorescence. The results obtained led to the conclusion that the fluorescence observed are due to the inhibition of the efflux of EB, as the compounds displayed almost no fluorescence by themselves and the same fluorescence as EB when applied in combination with it (results not shown). It can also be noted that some compounds present negative RFI values, which means that their relative fluorescence indexes are lower than that of the negative control, dimethyl sulfoxide (DMSO, 1% *v/v*), and were considered ineffective for this purpose. Figure 2 shows the comparison between the effective compounds and the positive controls.

Generally, none of the enantiomeric pairs showed activity for the same pump being the efflux pump inhibitory effect enantioselective. Noteworthy, the achiral precursors **5**–**7**, **9**, **10**, and **12** did not display favorable results for this activity. 

Considering these results, a possible binding site was investigated for these compounds, using PyMol program for visual inspection. According to the initial docking studies performed (Table 1), compound **(*R*,*R*)-8** was visualized in the BCR of the NorA homology model, whereas compounds **(*S*,*S*)-8**, **(*R*)-17** and **(*R*,*S*)-18** were visualized in the SBS of the AcrB portion. 

**Figure 3:**
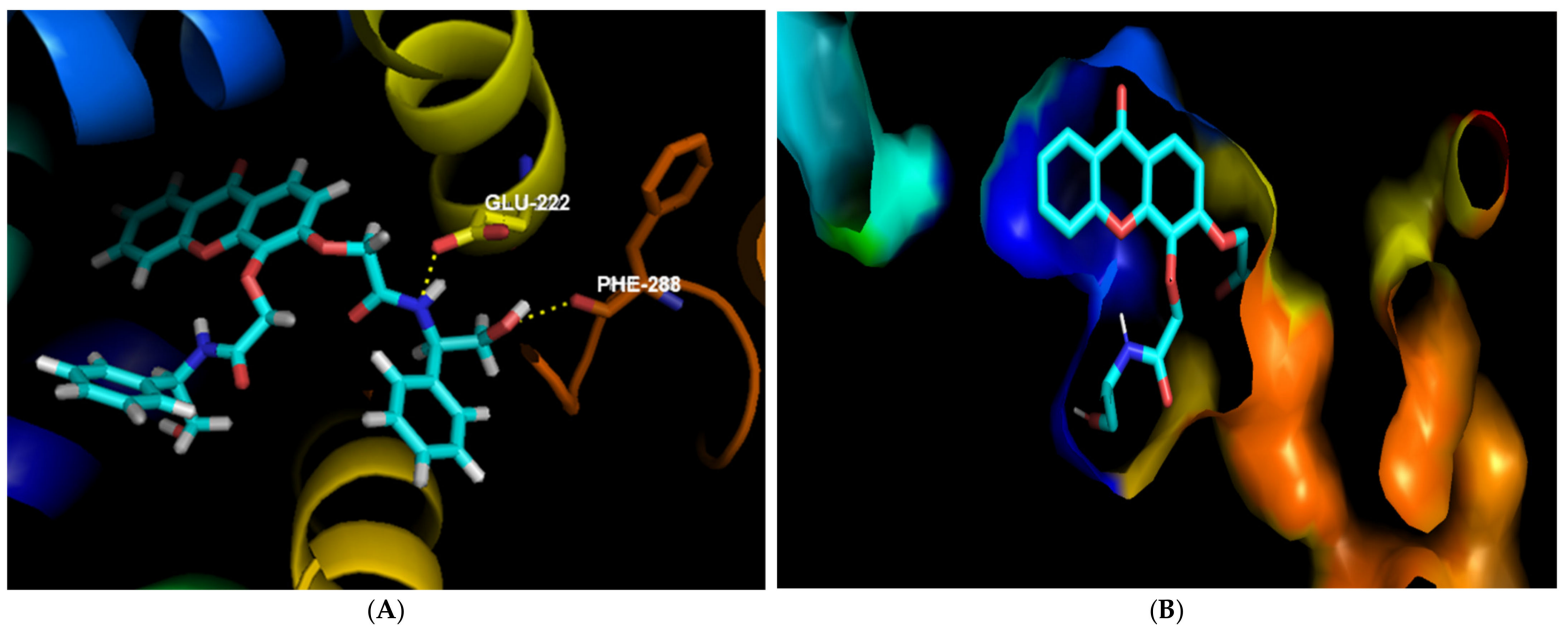

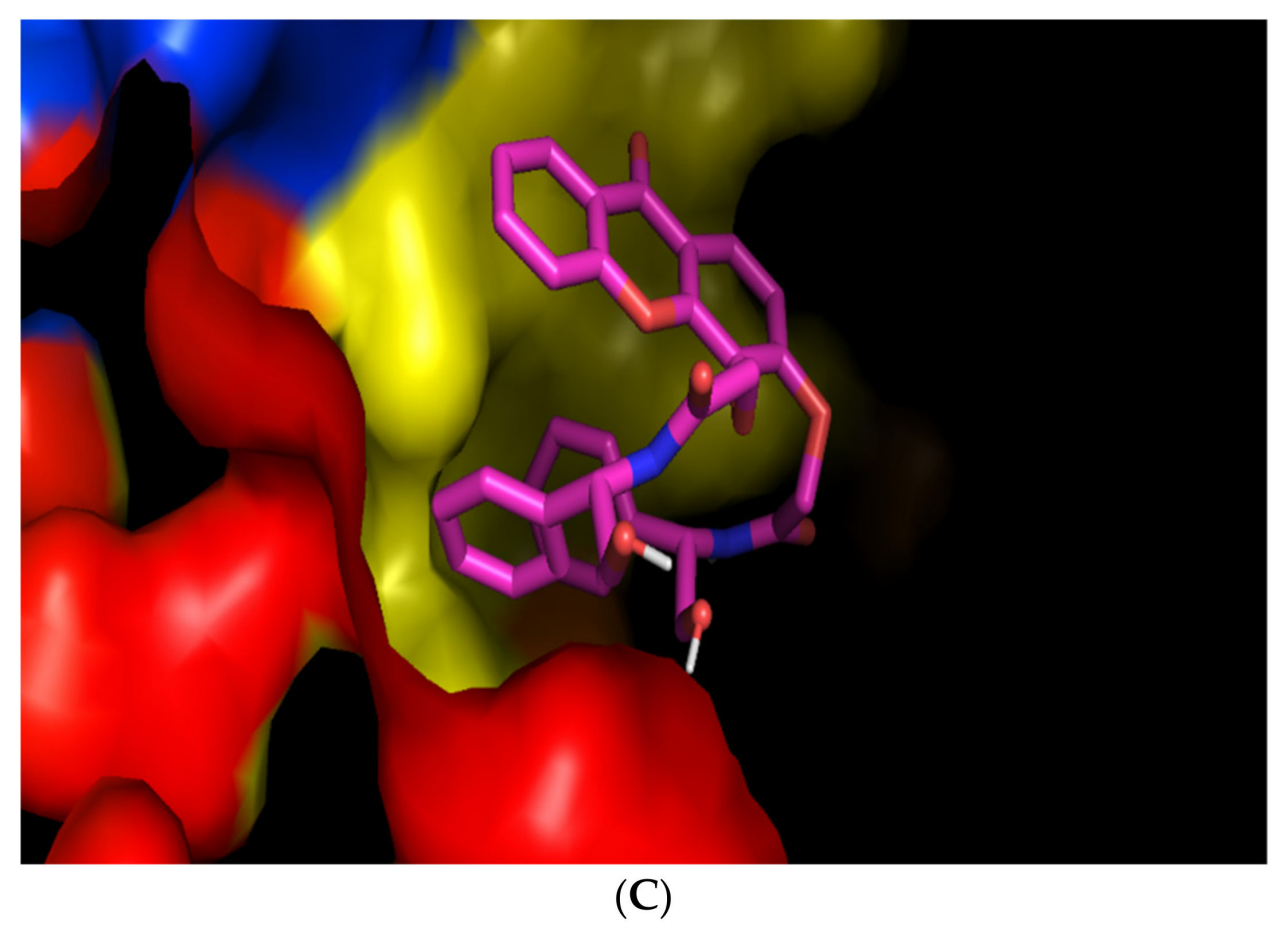
Molecular visualization of **(*R*,*R*)-8** (blue) and **(*S*,*S*)-8** (pink) in the BCR of the homology model of NorA; (**A**) interaction of **(*R*,*R*)-8** with key residues; (**B**) surface view of **(*R*,*R*)-8** inside a pocket; (**C**) visualization of **(*S*,*S*)-8** at the surface of the homology model.

In the interactions of **(*R*,*R*)-8** in NorA, it can be seen that the substituent in the C3 position was predicted to play an important role in binding to this portion of the efflux system. The amide establishes a hydrogen bond with Glu-222, as did structurally-related thioxanthones previously tested [53], and the hydroxyl moiety can do the same with Phe-288, as can be seen in Figure 3A. Besides, this compound seems to fit perfectly in a pocket present in this site (Figure 3B). Interestingly, its enantiomer **(*S*,*S*)-8** was predicted to bind with the most affinity in a different site of the BCR, as the two phenyl groups seem to establish a π-π stacking, making the position of the molecule too hindered to enter the pocket where **(*R*,*R*)-8** is predicted to bind (Figure 3C).

**Figure 4:**
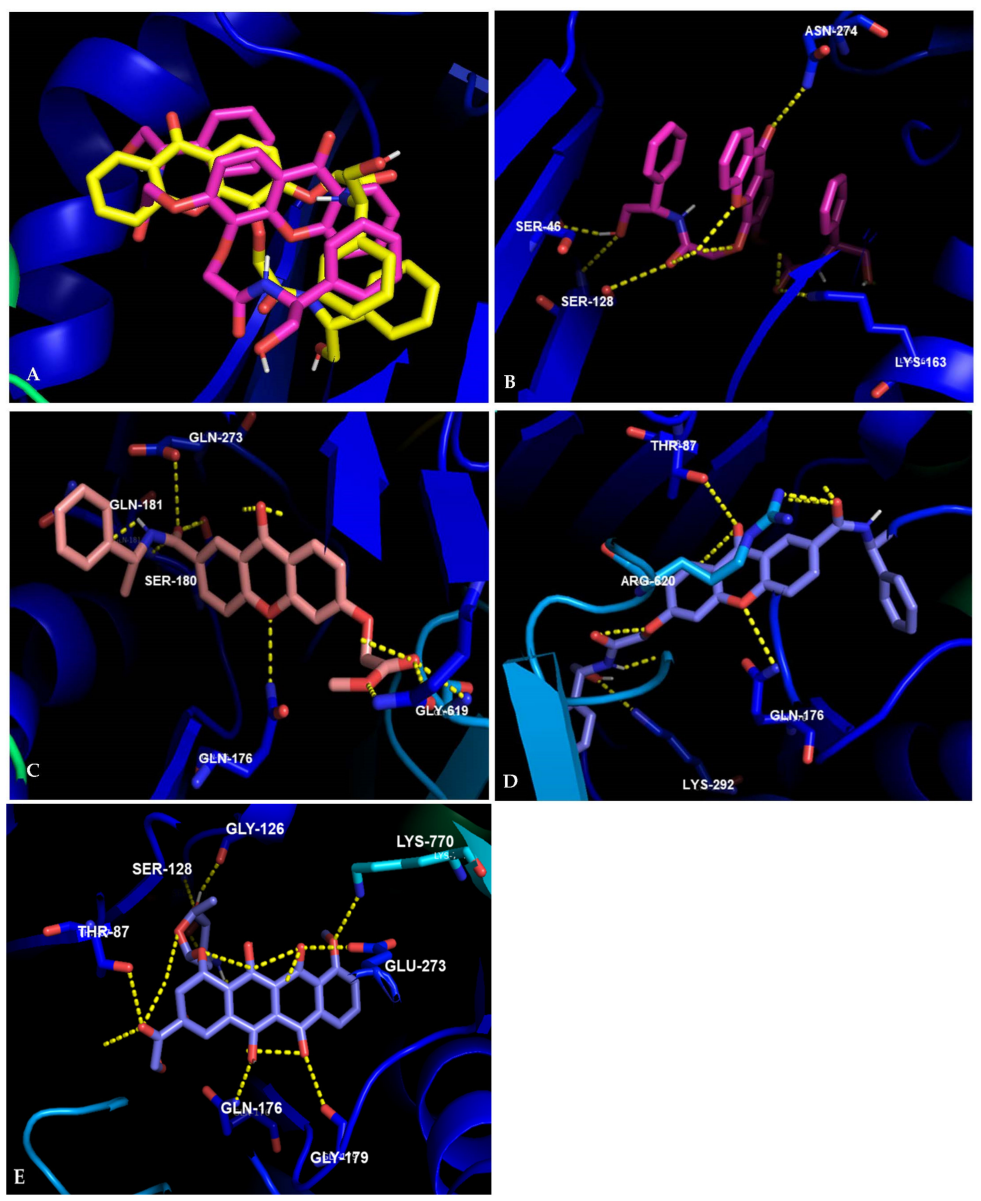
Molecular visualization of the effective compounds in the SBS of AcrB. (**A**) **(*R*,*R*)-8** (yellow) and **(*S*,*S*)-8** (pink) in the SBS of AcrB; (**B**) interactions of **(*S*,*S*)-8** with key residues; (**C**) interactions of **(*R*)-17** with key residues; (**D**) interactions of **(*R*,*S*)-18** with key residues; (**E**) interactions of doxorubicin with key residues.

Concerning the visualization in AcrB, the enantiomeric pair **8** is predicted to have a very different behavior than in the homology model of NorA, in accordance to experimental results. As can be seen in Figure 4A, compound **(*S*,*S*)-8**, which presented activity in the accumulation assay, spreads over a larger area than **(*R*,*R*)-8**. A deeper analysis of **(*S*,*S*)-8** shows interactions with Ser-46, Ser-128, Lys-163, Asn-274, and Arg-767 (Figure 4B). Previous studies showed that nitrogen-substituted thioxanthones also formed hydrogen bonds with Arg-274, and residues nearby the other described [32].

Compound **(*R*)-17** was predicted to interact with several residues previously described for xanthones and thioxanthones, such as Gln-89, Gln-176, and Gly-619 [32,53]. Additionally, this compound also interacts with Ser-180, Gln-181, and Gln-273 (Figure 4C). Lastly, compound **(*R*,*S*)-18** is predicted to establish hydrogen bonds mostly with residues previously described in other studies with (thio)xanthones, such as Thr-87. Gln-176 and Arg-620 [32,53]. The hydroxyl present in the compound can establish an additional hydrogen bond with Lys-292 (Figure 4D). Additionally, all the compounds presented non-polar interactions (not shown).

Doxorubicin is a known AcrB substrate, that has been co-crystallized with this portion of the AcrAB-TolC efflux system [54]. It possesses a tetracyclic scaffold, similar to the tricyclic scaffold of the xanthones presented herein, which led us to also analyze the predicted interactions between this drug and AcrB (Figure 4E), and were found to be similar to previously described [48,54]. Firstly, it can be noted that the carbonyl moiety on the carboxylic acid side chain of doxorubicin interacts with Thr-87. Compound **(*R*,*S*)-18** is also predicted to establish this interaction, specifically due to the carbonyl in the xanthone moiety. The amine in doxorubicin can establish a polar interaction with Ser-128, similar to what was described for the carbonyl in the amide present is compound **(*R*,*R*)-8**. Another residue that was predicted to be involved in the binding of doxorubicin, **(*R*)-17** and **(*R*,*S*)-18** was Gln-176, where a carbonyl of doxorubicin and the ether present in the xanthone scaffold were predicted to establish polar interactions. Doxorubicin also presents polar interactions with Gly-126, Gly-179, and Lys-770.

Although the results herein presented suggest the inhibition of efflux pumps, it cannot be ruled out that the compounds present other mechanisms that led to the results obtained. Further studies are warranted in order to clarify this subject.

### 2.5. Inhibition of Biofilm Formation and Quorum-Sensing

The chiral compounds were studied for their ability to inhibit the formation of biofilm and QS. As these mechanisms are related to the inhibition of efflux pumps [55,56], only the compounds that displayed inhibition of EB efflux in Section 2.4, **(*S*,*S*)-8**, **(*R*,*R*)-8, (*R*)-17**, and **(*R*,*S*)-18**, were tested. In fact, aside from the efflux of xenobiotics from the interior of the bacterial cell, efflux pumps induce the efflux of substances that are responsible for the formation of biofilms, and the transport of quorum-sensing molecules, e.g., acyl homoserine lactones (AHL). They are also involved in the regulation of genes involved in these phenomena and in the modulation of aggregation between bacteria and between bacteria and surfaces [57].

The biofilm formation assay was performed against *S. aureus* ATCC 29213 and *S. aureus* 272123. For this assay, reserpine was used as a positive control, as it is both an inhibitor of efflux pumps and biofilm [50,58]. The inhibition of biofilm formation, prevention of the adhesion or degradation was expressed in percentage (%) and was calculated based on the mean of the absorbance units. All the compounds were tested at 100 µM, as no compounds presented antibacterial activity at this concentration for the strains tested. The results obtained in this assay are depicted in Table 3.

All the compounds were more active in *S. aureus* 272123 than in the ATCC strain, as the compounds had no influence in the biofilm formation in the latter, although less effective than reserpine. Interestingly, compound **(*R*,*R*)-8**, which was the most active on biofilm inhibition, was also the only compound able to inhibit efflux pumps in *S. aureus* 272123, suggesting a possible related mechanism.

The ability of these compounds to inhibit QS was also investigated. For that, four Gram-negative bacteria were used: the sensor strain *Chromobacterium violaceum* CV026 (CV026), which was inoculated as a parallel line to *Sphingomonas paucimobilis* Ezf 10–17 (EZF), a producer strain of AHL, and the AHL producers *Serratia marcescens* AS-1 and *Chromobacterium violaceum* wild-type 85 (wt85), which were inoculated as single lines. Promethazine (PMZ) was used as positive control, and the inhibition of QS was observed as the reduction in pigment production and measured in millimeters (mm) [59,60]. The results are present in Table 3.

Interestingly, only the compounds corresponding to the enantiomeric pair **8** displayed inhibition of QS in the system EZF + CV026. Even though bacteria of the *Chromobacterium* species have been proven to present RND efflux pumps [61], a causative relationship between the inhibition of these pumps and QS could not be established at this point, but is a topic that deserves deeper studies.

## 3. Materials and Methods

All reagents and solvents used for the synthesis were purchased from Sigma Aldrich (Sigma-Aldrich Co. Ltd., Gillinghan, UK) and were not subject to any purification process. A rotary evaporator Buchi Rotavap R-210 and a Buchi Heating Bath B-491 were used to evaporate the solvents under reduced pressure, with a Huber mini-chiller ensuring refrigeration. Thin-layer chromatography (TLC) was carried out to monitor the reactions, on precoated plates with 0.2 mm of thickness using Merck silica gel 60 (G/UV_254_) using suitable mobile phases. The compounds herein described were easily detectable at 254 nm or 365 nm.

The synthesized products were purified by flash column chromatography, using silica gel 60 (0.040–0.063 mm, Acros Organics, Geel, Belgium). Melting points (mp) were measured in a Köfler microscope (Wagner and Munz, Munich, Germany) and were uncorrected. For structure elucidation, ^1^H- and ^13^C-nuclear magnetic resonance (NMR) spectra were recorded at room temperature on a Bruker Avance 300 spectrometer (300.13 MHz for ^1^H and 75.47 MHz for ^13^C, Bruker Biosciences Corporation, Billerica, MA, USA), at the Department of Chemistry, University of Aveiro. The samples were solubilized in DMSO-*d*_6_ or CDCl_3_. (Aldrich, Steinheim, Germany). Chemical shifts are shown in δ (ppm) values relative to tetramethylsilane (TMS), used as an internal reference. Coupling constants are reported in hertz (Hz). The assignments of ^13^C-NMR were made by bidimensional heteronuclear single quantum coherence (HSQC) and heteronuclear multiple bond correlation (HMBC) NMR experiments (long-range C, H coupling constants were optimized to 7 Hz) or by comparison with the assignments of similar molecules. High-resolution mass spectroscopy (HRMS) spectra were measured on a LTQ Orbitrap XL hybrid mass spectrometer (Thermo Fischer Scientific, Bremen, Germany) at CEMUP, University of Porto, Portugal. The optical rotations were recorded on a Polartronic Universal polarimeter (ADP 410 polarimeter).

The solvents used for cLC analysis (CH_3_CN, CH_3_OH, 2-propanol, *n*-hexane, and acetic acid) were of high-performance liquid chromatography (HPLC) grade and purchased from Sigma-Aldrich (Sigma-Aldrich Co. Ltd., Gillinghan, UK). The stock solutions of the chiral derivatives of xanthone were prepared by dissolution in CH_3_CH_2_OH at a concentration of 1 mg/mL and further diluted to a concentration of 10 μg/mL. Aliquots of each enantiomer were mixed to obtain working solutions. The e.r. measurements were performed with the stock solutions of each enantiomer diluted at a concentration of 20 μg/mL.

The culture media used in the experiments were purchased: the cation-adjusted Mueller–Hinton broth (MHB II) was purchased from Sigma-Aldrich, St. Louis, MO, USA and Biokar Diagnostics, Allone, Beauvais, France, the Luria-Bertani broth (LB-B) from Sigma, St. Louis, MO, USA, Tryptic Soy broth (TSB) was bought from Scharlau Chemie S.A., Barcelona, Tryptic-Soy agar (TSA) was purchased from Biokar Diagnostics, Allone, Beauvais, France), Sabouraud Dextrose Agar (SDA) from bio-Mérieux, Marcy L’Etoile, France, RPMI-1640 broth medium from Biochrom AG, Berlin, Germany, which was buffered with 3-(*N*-morpholino) propanesulfonic acid (MOPS), purchased from Sigma-Aldrich, St. Louis, MO, USA, to pH 7.0. The modified Luria–Bertani agar (LB*-A) was prepared in-house, according to the formula: 1.0 g yeast extract (Merck, Darmstadt, Germany), 10.0 g tryptone (Biolab, Budapest, Hungary), 10.0 g NaCl (Molar Chemicals, Halásztelek, Hungary), 1.0 g K_2_HPO_4_ (Biolab, Budapest, Hungary), 0.3 g MgSO_4_ × 7H_2_O (Reanal, Budapest, Hungary), 5 mL Fe-EDTA stock solution and 20.0 g of bacteriological agar (Molar Chemicals, Halásztelek, Hungary) per 1 L of media.

Dimethyl sulfoxide (DMSO), phosphate-buffered saline (PBS; pH 7.4), ethidium bromide (EB), reserpine, carbonyl cyanide 3-chlorophenylhydrazone (CCCP), promethazine (PMZ) and crystal violet (CV) were purchased from Sigma-Aldrich Chemie GmbH (Steinheim, Germany). The antibiotic cefotaxime (CTX) was purchased from Duchefa Biochemie (Haarlem, The Netherlands). Bacteria were purchased from ATCC.

### 3.1. Chemistry

The syntheses of compounds **1**–**8** [62] and **13**–**15** [44] were previously reported. Compound **1** was obtained through a benzophenone route, as described previously. After its demethylation different products could be obtained. The main product was the 3-methoxy-4-hydroxy-9*H*-xanthen-9-one (**2**), although the formation of secondary products, 3-hydroxy-4-methoxy-9*H*-xanthen-9-one (**4**) and 3,4-dihydroxy-9*H*-xanthen-9-one (**3**) occurred [62].

Starting from **3**, the derivatives **6**, **7*,*** and **8** were obtained, as previously described [62], with yields of 95%, 81%, and 57%, respectively. The synthesis of compounds **9–12** will be detailed in Section 3.1.1, Section 3.1.2, Section 3.1.3 and Section 3.1.4. The synthesis of derivatives **16**–**18** was accomplished using **13** as a starting material, which was obtained via Ullmann reaction, which then yielded compounds **14** (50%) and **15** (95%), following the procedure in [44]. Detailed descriptions can be found in Section 3.1.5, Section 3.1.6 and Section 3.1.7.

#### 3.1.1. Synthesis of Methyl 2-[(3-Methoxy-9-oxo-9*H*-xanthen-4-yl)oxy]acetate (**9**)

Methyl 2-[(3-methoxy-9-oxo-9*H*-xanthen-4-yl)oxy]acetate (**9**) was prepared from 3-methoxy-4-hydro-9*H*-xanthen-9-one (**2**), using the same method as previously described for 2′-[(9-oxo-9*H*-xanthene-3,4-diyl)bis(oxy)]diacetate (**6**), adjusting the amount of K_2_CO_3_ and BrCH_2_COOCH_3_ according to the number of hydroxyl groups of the chemical substrate and the stoichiometry of the reaction [62]. The product was purified by *flash* chromatography (*n*-hexane/ethyl acetate in gradient) affording **9** as white solid.

Yield: 40%. m.p.: 135–138 °C. IR ν_max_ (cm^−1^) (KBr): 1750.8, 1603.4, 1509.1, 1464.6, 1388.0, 1288.2, 1216.3, 1033.5, 819.5, 767.6. ^1^H NMR (300.13 MHz, DMSO-*d*_6_) δ: 8.17 (dd, *J* = 7.9, 1.7 Hz, 1H, H-8), 7.95 (d, *J* = 9.0 Hz, 1H, H-1), 7.85 (ddd, *J* = 8.4, 7.2, 1.7 Hz, 1H, H-6), 7.63 (dd, *J* = 8.4, 1.7 Hz, 1H, H-5), 7.47 (ddd, *J* = 7.9, 7.2, 1.7 Hz, 1H, H-7), 7.27 (d, *J* = 9.0 Hz, 1H, H-2), 4.85 (s, 2H, OC**H_2_**), 3.73 (s, 3H, OC**H_3_**), 3.36 (s, 3H, COOC**H_3_**). ^13^C NMR (75.47 MHz, DMSO-*d*_6_) δ: 175.2 (C-9), 169.1 (**C**OOCH_3_), 156.7 (C-3), 155.5 (C-4), 149.4 (C-1), 135.3 (C-10a), 134.1 (C-4a), 125.9 (C-2), 124.4 (C-6), 121.7 (C-8), 120.8 (C-7), 118.1 (C-5), 115.8 (C-8a), 109.7 (C-9a), 69.3 (O**C**H_2_), 56.6 (O**C**H_3_), 51.7 (COO**C**H_3_) (Figure S1, Supplementary Data). HRMS (ESI+): *m*/*z* [C_17_H_14_O_6_ + H]+ calcd. for [C_17_H_15_O_6_]: 315.08687; found 315.08569 (Figure S2, Supplementary Data).

#### 3.1.2. Synthesis of 2-[(3-Methoxy-9-oxo-9*H*-xanthen-4-yl)oxy]acetic Acid (**10**)

Compound **10** was prepared from **9**, using the same method as described for 2,2′-[(9-oxo-9*H*-xanthene-3,4-diyl)bis(oxy)]diacetic acid (**7**), adjusting the volume of the solution of 5M NaOH according to the number of acetate groups of the chemical substrate and the stoichiometry of the reaction [62], yielding 2-[(3-methoxy-9-oxo-9*H*-xanthen-4-yl)oxy]acetic acid (**10**) as white solid.

Yield: 97%. m.p.: 205–208 °C. IR ν_max_ (cm^−1^) (KBr): 3204.0, 2925.0, 1758.6, 1604.1, 1585.2, 1467.8, 1290.4, 1229.6, 1033.1, 819.1, 766.6. ^1^H NMR (300.13 MHz, DMSO-*d*_6_) δ: 12.9 (s, 1H, COO**H**), 8.17 (dd, *J* = 8.0, 1.7 Hz, 1H, H-8), 7.90 (d, *J* = 9.0 Hz, 1H, H-1), 7.80 (ddd, *J* = 8.6, 7.2, 1.7 Hz, 1H, H-6), 7.64 (dd, *J* = 8.6, 1.7 Hz, 1H, H-5), 7.48 (ddd, *J* = 8.0, 7.2, 1.7 Hz, 1H, H-7), 7.27 (d, *J* = 9.0 Hz, 1H, H-1), 4.76 (s, 2H, OC**H**_2_), 3.97 (s, 3H, OC**H**_3_). ^13^C NMR (75.47 MHz, DMSO-*d*_6_) δ: 175.3 (C-9), 170.1 (COO**H**), 156.7 (C-3), 155.5 (C-4), 149.4 (C-1), 135.3 (C-10a), 134.3 (C-4a), 125.9 (C-2), 124.4 (C-6), 121.5 (C-8), 120.8 (C-7), 118.2 (C-5), 115.9 (C-8a), 109.8 (C-9a), 69.2 (OC**H**_2_), 56.6 (OC**H**_3_) (Figure S3, Supplementary Data). HRMS (ESI+): *m*/*z* [C_16_H_12_O_6_ + H]+ calcd. for [C_16_H_13_O_6_]: 301.07122; found 301.07191 (Figure S4, Supplementary Data).

#### 3.1.3. Synthesis of (*S*)-2-[(3-Methoxy-9-oxo-9*H*-xanthen-4-yl)oxy]-*N*-(1-phenylethyl)acetamide [**(*S*)-11**] and (*R*)-2-[(3-Methoxy-9-oxo-9*H*-xanthen-4-yl)oxy]-*N*-(1-phenylethyl)acetamide [**(*R*)-11**]

The enantiomeric pair (*S*)-2-[(3-methoxy-9-oxo-9*H*-xanthen-4-yl)oxy]-*N*-(1-phenylethyl)acetamide [**(*S*)-11**] and (*R*)-2-[(3-methoxy-9-oxo-9*H*-xanthen-4-yl)oxy]-*N*-(1-phenylethyl)acetamide [**(*R*)-11**] was prepared from the carboxyl compound **10**, using the same method as described for compound **8** [62], and the chiral reagents (*S*)-(−)-(*α*)-methylbenzylamine and (*R*)-(+)-(*α*)-methylbenzylamine, respectively. The final products were obtained as white solid.

(*S*)-2-[(3-Methoxy-9-oxo-9*H*-xanthen-4-yl)oxy]-*N*-(1-phenylethyl)acetamide [**(*S*)-11**]: Yield: 45%. m.p.: 164–167 °C. [α]_D_^25 °C^ + 48.2 (*c* = 0.83 × 10^−3^ gmL^−1^ in CH_3_OH). e.r: > 99% (k: 1.40) (Figure S5, Supplementary Data). 

(*R*)-2-[(3-Methoxy-9-oxo-9*H*-xanthen-4-yl)oxy]-*N*-(1-phenylethyl)acetamide [**(*R*)-11**]: Yield: 50%. m.p.: 165–167 °C. [α]_D_^25 °C^ − 48.4 (*c* = 0.83 × 10^−3^ gmL^−1^ in CH_3_OH). e.r: > 99% (k: 0.78) (Figure S5, Supplementary Data). 

IR ν_max_ (cm^−1^) (KBr): 3348.1, 1665.1, 1604.8, 1587.5, 1466.9, 1289.1, 1230.2, 1023.4, 817.7, 769.1. ^1^H NMR (300.13 MHz, DMSO-*d*_6_) δ: 8.38 (d, *J* = 8.2 Hz, 1H, N**H**), 8.17 (dd *J* = 8.0, 1.8 Hz, 1H, H-8), 7.96 (d, *J* = 9.0 Hz, 1H, H-1), 7.82 (ddd, *J* = 8.7, 7.1, 1.8 Hz, 1H, H-6), 7.50 (dd, *J* = 8.7, 1.8 Hz, 1H, H-5), 7.46 (ddd, *J* = 8.0, 7.1, 1.8 Hz, 1H, H-7), 7.45 – 7.27 (m, 5H, H-2″ to H-6″), 7.26 (d, *J* = 9.0 Hz, 1H, H-2), 5.10 (q, *J* = 8.2 Hz, 1H, H-1′), 4.65 (s, 2H, OC**H**_2_), 3.93 (s, 3H, OC**H**_3_), 1.48 (d, *J* = 8.2 Hz, 3H, H-2′). ^13^C NMR (75.47 MHz, DMSO-*d*_6_) δ: 175.2 (C-9), 167.1 (**C**ONH), 156.6 (C-3), 155.4 (C-4), 149.3 (C-1), 135.2 (C-10a), 134.7 (C-4a), 128.4 (C-1′’), 126.9 (C-3″ to C-5′’), 126.1 (C-2″ and C-6′’), 125.9 (C-2), 124.4 (C-6), 121.9 (C-8), 120.8 (C-7), 118.2 (C-5), 109.6 (C-9a), 72.4 (OC**H**_2_), 56.6 (OC**H**_3_), 47.7 (C-1′), 22.1 (C-2′) (Figure S6, Supplementary Data). HRMS (ESI+): *m*/*z* [C_24_H_21_NO_5_ + H]+ calcd. for [C_24_H_22_NO_5_]: 404.14980; found 404.14816 (Figure S7, Supplementary Data).

#### 3.1.4. Synthesis of 4-(2-Hydroxyethoxy)-3-methoxy-9*H*-xanthen-9-one (**12**)

Starting from compound **2**, compound **12** was synthesized through an adaptation of a protocol previously described for Williamson ether synthesis [62]. Compound **2** (0.23 g, 0.95 mmol) was dissolved in anhydrous acetone, and NaH (0.10 g, 4.17 mmol) and BrCH_2_CH_2_OH (0.30 mL, 4.23 mmol) were added. The mixture was kept under reflux and magnetic stirring for 24 h. Then, the organic solvent was evaporated under reduced pressure, and the crude product was dissolved in ethyl acetate. This solution was washed with a 0.5 M NaOH solution (3 × 100 mL) and H_2_O (2 × 50 mL). The organic layer was dried with anhydrous NaSO_4_, filtered, and the organic solvent was evaporated under reduced pressure affording a yellow solid.

Yield: 80%. m.p.: 137–140 °C. IR ν_max_ (cm^−1^) (KBr): 3423.2, 1602.0, 1467.0, 1290.5, 1224.5, 1068.1, 905.5, 823.5, 760.4. ^1^H NMR (300.13 MHz, CDCl_3_) δ: 8.33 (dd, *J* = 6.0, 1.3 Hz, 1H, H-8), 8.12 (d, *J* = 6.8 Hz, 1H, H-1), 7.84 (m, 1H, H-6), 7.53 (dd, *J* = 6.3, 0.9 Hz, 1H, H-5), 7.39 (ddd, *J* = 6.0, 5.3, 0.8 Hz, 1H, H-7), 7.03 (d, *J* = 6.8 Hz, 1H, H-2), 4.33 (t, *J* = 3.3 Hz, 2H, OC**H**_2_), 4.05 (s, 1H, OH), 4.03 (s, 3H, OC**H**_3_), 3.90 (t, *J* = 3.5 Hz, 2H, C**H**_2_OH). ^13^C NMR (75.47 MHz, CDCl_3_) δ: 175.3 (C-9), 157.5 (C-3), 155.5 (C-4), 150.0 (C-1), 135.2 (C-10a), 135.1 (C-4a), 125.8 (C-2), 124.2 (C-6), 121.4 (C-8), 120.8 (C-7), 118.2 (C-5), 115.9 (C-8a), 109.7 (C-9a), 75.1 (OC**H**_2_), 60.4 (C**H**_2_OH), 56.5 (OC**H**_3_) (Figure S8, Supplementary Data). HRMS (ESI+): *m*/*z* [C_16_H_14_O_5_ + H]+ calcd. for [C_16_H_15_O_5_]: 287.09195; found 287.09042 (Figure S9, Supplementary Data).

#### 3.1.5. Synthesis of (*S*)-2-{[9-oxo-7-((1-Phenylethyl)carbamoyl)-9*H*-xanthen-3-yl]oxy}acetate [**(*S*)-16**] and of (*R*)-2-{[9-oxo-7-((1-Phenylethyl)carbamoyl)-9*H*-xanthen-3-yl]oxy}acetate [**(*R*)-16**]

Starting from compound **(*S*)-15** or **(*R*)-15**, a reaction was carried out under anhydrous conditions and nitrogen atmosphere according to the described procedure [63] to afford an orange oil. Then, the synthesis of compounds **(*S*)-16** and **(*R*)-16** was carried out using the same method as described, previously, for 2′-[(9-oxo-9*H*-xanthene-3,4-diyl)bis(oxy)]diacetate (**6**), adjusting the amount of K_2_CO_3_ and BrCH_2_COOCH_3_ according to the number of hydroxyl groups of the chemical substrate and the stoichiometry of the reaction [62], affording **(*S*)-16** and **(*R*)-16** as white solid.

(*S*)-2-{[9-oxo-7-((1-Phenylethyl)carbamoyl)-9*H*-xanthen-3-yl]oxy}acetate [**(*S*)-16**]: Yield: 80%. m.p.: > 330 °C. [α]_D_^25 °C^ + 686.0 (*c* = 0.86 × 10^−3^ gmL^−1^ in CH_3_OH). e.r: > 99% (k: 1.63) (Figure 1). 

(*R*)-2-{[9-oxo-7-((1-Phenylethyl)carbamoyl)-9*H*-xanthen-3-yl]oxy}acetate [**(*R*)-16**]: Yield: 75%. m.p.: > 330 °C. [α]_D_^25 °C^ − 686.0 (*c* = 0.86 × 10^−3^ gmL^−1^ in CH_3_OH). e.r: > 99% (k: 0.52) (Figure 1). 

IR ν_max_ (cm^−1^) (KBr): 3431.7, 3295.6, 1759.3, 1648.4, 1617.9, 1540.7, 1479.7, 1348.7, 1213.5, 1067.7, 828.6, 781.6. ^1^H NMR (300.13 MHz, DMSO-*d*_6_) δ: 9.20 (d, *J* = 7.8 Hz, 1H, CON**H**), 8.76 (d, *J* = 2.3 Hz, 1H, H-1), 8.33 (dd, *J* = 8.8, 2.3 Hz, 1H, H-3), 8.15 (d, *J* = 8.9 Hz, 1H, H-8), 7.71 (d, *J* = 8.8 Hz, 1H, H-4), 7.45–7.26 (d,t,d, *J* = 7.5 Hz, 5H, H-1″ to H-6″), 7.23 (d, *J* = 2.4 Hz, 1H, H-5), 5.21 (m, 1H, H-1′), 5.07 (s, 2H, OC**H**_2_), 3.74 (s, 3H, COOC**H**_3_), 1.52 (d, *J* = 7.8 Hz, 3H, H-2′). ^13^C NMR (75.47 MHz, DMSO-*d*_6_) δ: 174.9 (C-9), 168.6 (**C**ONH), 164.1 (**C**OOCH_3_), 163.4 (C-6), 157.3 (C-10a), 157.2 (C-4a), 144.8 (C-1″), 134.1 (C-3), 130.4 (C-2), 128.3 (C-3″ to C-5″), 126.7 (C-8), 126.1 (C-2″ and C-6″), 125.5 (C-1), 118.2 (C-8a), 114.1 (C-7), 101.8 (C-5), 65.1 (O**C**H_2_), 52.1 (O**C**H_3_), 48.7 (C-1′), 22.2 (C-2′) (Figure S10, Supplementary Data). HRMS (ESI+): *m*/*z* [C_24_H_19_NO_6_ + H]+ calcd. for [C_24_H_20_NO_6_]: 432.14471; found 432.14410 (Figure S11, Supplementary Data).

#### 3.1.6. Synthesis of (*S*)-2-{[9-oxo-7-((1-Phenylethyl)carbamoyl)-9*H*-xanthen-3-yl]oxy}acetic Acid [**(*S*)-17**] and of (*R*)-2-{[9-oxo-7-((1-Phenylethyl)carbamoyl)-9*H*-xanthen-3-yl]oxy}acetic Acid [**(*R*)-17**]

The esters **(*S*)-16** and **(*R*)-16** were hydrolysed to the acetic acid derivatives **(*S*)-17** and **(*R*)-17** through the method previously described for compound **7** [62], furnishing a white solid. 

(*S*)-2-{[9-oxo-7-((1-phenylethyl)carbamoyl)-9*H*-xanthen-3-yl]oxy}acetic acid [**(*S*)-17**]: Yield: 51%. m.p.: > 330 °C. [α]_D_^25 °C^ + 450.0 (*c* = 0.75 × 10^−3^ gmL^−1^ in CH_3_OH). e.r: > 98% (k: 0.56) (Figure S12, Supplementary Data).

(*R*)-2-{[9-oxo-7-((1-phenylethyl)carbamoyl)-9*H*-xanthen-3-yl]oxy}acetic acid [**(*R*)-17**]: Yield: 71%. m.p.: > 330 °C. [α]_D_^25 °C^ − 450.0 (*c* = 0.75 × 10^−3^ gmL^−1^ in CH_3_OH). e.r: > 99% (k: 0.38) (Figure S12, Supplementary Data).

IR ν_max_ (cm^−1^) (KBr): 3306.9, 3082.7, 2977.7, 1718.6, 1664.0, 1619.8, 1552.4, 1480.6, 1236.6, 1065.8, 830.9, 779.2. ^1^H NMR (300.13 MHz, DMSO-*d*_6_) δ: 9.19 (d, *J* = 7.9 Hz, 1H, CON**H**), 8.74 (d, *J* = 2.3 Hz, 1H, H-1), 8.31 (dd, *J* = 8.8, 2.3 Hz, 1H, H-3), 8.14 (d, *J* = 9.0 Hz, 1H, H-8), 7.72 (d, *J* = 8.8 Hz, 1H, H-4), 7.44–7.24 (d,t,d, *J* = 7.5 Hz, 5H, H-1′’ to H-6′’), 7.17 (d, *J* = 2.1 Hz, 1H, H-5), 7.12 (dd, *J* = 9.0, 2.1 Hz, 1H, H-7), 5.21 (m, 1H, H-1′), 4.94 (s, 2H, OC**H**_2_), 1.52 (d, *J* = 7.9 Hz, 3H, H-2′). ^13^C NMR (75.47 MHz, DMSO-*d*_6_) δ: 174.8 (C-9), 165.4 (**C**ONH), 164.1 (**C**OOH), 163.6 (C-6), 157.3 (C-10a), 157.2 (C-4a), 144.8 (C-1″), 134.0 (C-3), 130.3 (C-2), 128.2 (C-3″ to C-5″), 126.6 (C-8), 126.1 (C-2″ and C-6″), 125.4 (C-1), 118.1 (C-8a), 114.0 (C-7), 101.6 (C-5), 65.0 (OC**H**_2_), 48.7 (C-1′), 22.1 (C-2′) (Figure S13, Supplementary Data). HRMS (ESI+): *m*/*z* [C_24_H_19_NO_6_ + H]+ calcd. for [C_24_H_20_NO_6_]: 418.12906; found 418.12802 (Figure S14, Supplementary Data).

#### 3.1.7. Synthesis of 6-{2-[((*R*)-2-Hydroxy-1-phenylethyl)amino]-2-oxoethoxy}-9-oxo-*N*-[(*S*)-1-phenylethyl]-9*H*-xanthene-2-carboxamide [**(*R,S*)-18**] and of 6-{2-[((*S*)-2-Hydroxy-1-phenylethyl)amino]-2-oxoethoxy}-9-oxo-*N*-[(*R*)-1-phenylethyl]-9*H*-xanthene-2-carboxamide [**(*S,R*)-18**]

Compounds **(*R,S*)-18** and **(*S,R*)-18** were synthetized according to the method previously described for compound **8** [62], being obtained as white solid, using the enantiomers (*S*)-(+)-2-phenylglycinol and (*R*)-(−)-2-phenylglycinol, respectively.

6-{2-[((*R*)-2-Hydroxy-1-phenylethyl)amino]-2-oxoethoxy}-9-oxo-*N*-[(*S*)-1-phenylethyl]-9*H*-xanthene-2-carboxamide [**(*R,S*)-22**]: Yield: 65%. m.p.: > 330 °C. [α]_D_^25 °C^ − 211.3 (*c* = 0.71 × 10^−3^ gmL^−1^ in CH_3_OH). e.r: > 99% (k: 2.58) (Figure S15, Supplementary Data). 

6-{2-[((*S*)-2-Hydroxy-1-phenylethyl)amino]-2-oxoethoxy}-9-oxo-*N*-[(*R*)-1-phenylethyl]-9*H*-xanthene-2-carboxamide [**(*S,R*)-22**]: Yield: 63%. m.p.: > 330 °C. [α]_D_^25˚C^ + 211.0 (*c* = 0.71 × 10^−3^ gmL^−1^ in CH_3_OH). e.r: > 99% (k: 0.47) (Figure S15, Supplementary Data).

IR ν_max_ (cm^−1^) (KBr): 3261.5, 1654.3, 1616.5, 1471.4, 1222.4, 1140.4, 1060.4, 911.8, 763.0. ^1^H NMR (300.13 MHz, DMSO-*d*_6_) δ: 9.21 (d, *J* = 8.1 Hz, 1H, CON**H**C-1′), 8.78 (d, *J* = 2.4 Hz, 1H, H-1), 8.68 (d, *J* = 8.1 Hz, 1H, CON**H**C-1′′′), 8.33 (dd, *J* = 9.0, 2.4 Hz, 1H, H-3), 8.15 (d, *J* = 9.0 Hz, 1H, H-8), 7.72 (d, *J* = 9.0 Hz, 1H, H-4), 7.46–7.26 (d,t,d, *J* = 7.5 Hz, 10H, H-1′′ to H-6″ and H-1′′′′ to H-6′′′′, 7.23 (d, *J* = 2.5 Hz, 1H, H-5), 7.13 (dd, *J* = 9.0, 2.5 Hz, 1H, H-7), 5.22 (m, 1H, H-1′), 5.05 (s, 1H, O**H**), 4.92 (m, 1H, H-1′′′), 4.82 (s, 2H, OC**H**_2_), 3.61 (m, 2H, C**H**_2_OH), 1.52 (d, *J* = 8.1 Hz, 3H, H-2′). ^13^C NMR (75.47 MHz, DMSO-*d*_6_) δ: 174.8 (C-9), 166.5 (**C**ONHC-1′), 164.1 (CONH**C**-1′), 163.7 (C-6), 157.2 (C-10a), 157.1 (C-4a), 144.8 (C-1′′), 140.8 (C-1′′′′), 134.1 (C-3), 130.4 (C-2), 128.3 (C-3′′ to C-5′′), 128.1 (C-1′′′), 126.9 (C-3′′′′ to C-5′′′′), 126.6 (C-8), 126.1 (C-2′′, C-6′′, C-2′′′′ and C-6′’’’), 125.4 (C-1), 118.2 (C-8a), 115.3 (C-4), 114.5 (C-7), 101.7 (C-6), 64.4 (O**C**H_2_), 55.0 (**C**H_2_OH), 48.7 (C-1′), 22.2 (C-2′) (Figure S16, Supplementary Data). HRMS (ESI+): *m*/*z* [HRMS (ESI+): *m*/*z* [C_32_H_28_N_2_O_6_ + H]+ calcd. for [C_32_H_29_N_2_O_6_]: 537.20256; found 537.20142 (Figure S17, Supplementary Data).

### 3.2. Chiral Liquid Chromatography

The cLC analyses of the enantiomeric purity were performed on a Jasco model 880-PU Intelligent HPLC pump (JASCO Corporation, Tokyo, Japan), equipped with a JASCO model 880-30 solvent mixer involving an 875-UV intelligent UV-vis detector and a 7125 injector (Rheodyne LCC., Rohnert Park, California) fitted with a 20 μL loop. The data were handled on ChromNAV Chromatography Data System (version 1.19.1) from JASCO Corporation (Tokyo, Japan). The chromatographic column used was (*S*,*S*)-Whelk-O1^®^ (25 cm × 4.6 mm i.d., 5 µm particle size, 100-Å pore size) from Regis Technologies, Inc. (Morton Grove, IL, USA. The mobile phase was degassed in an ultrasonic bath for 15 min after preparation in a volume/volume ratio. Analyses were performed at 25 ± 2 °C in isocratic mode. The flow rate used was 0.5 mL/min, and the working pressure was between 30 and 35 bar. The chromatograms were monitored by UV at a wavelength of 254 nm. The sample injections (10 µL) were carried out in triplicate. The column void time (t_0_) was considered to be equal to the peak of the solvent front and was taken from each particular run. The e.r. was determined by the relative percentages of the peak areas according to e.r. (%) = 100 × ([R]/([R] + [S]) or 100 × ([S]/([S] + [R]), where [S] and [R] are the area of the peak of each enantiomer [64].

### 3.3. Docking Studies

The crystal structures of the AcrB (PDB: 4DX5) [54], AcrA (PDB: 2F1M) [65], and TolC (PDB: 1EK9) [66] portions of the AcrAB-TolC bacterial efflux system, downloaded from the protein databank (PDB) [67], were used for this study. The structures of the known AcrAB-TolC inhibitors D13-9001, doxorubicin, MBX-3132, minocycline, and phenyl-arginyl-β-naphthylamide, along with the structures of the tested compounds were drawn with ChemDraw (PerkinElmer Informatics, MA, USA) and minimized using ArgusLab. Docking was carried out using AutoDock Vina (Scripps, La Jolla, CA, USA) [68] in the sites described in [47,48]. Since the crystal structure of NorA efflux pump is not available, a homology model was prepared. The model was generated using the Swiss Model server [69] and the sequence was deposited in Uniprot (Q5HHX4) [70], using the EmrD pump from *Escherichia coli* (PDB: 2GFP) as the homolog, as described previously [49]. The top 9 poses were collected for each molecule, and the lowest docking score value was associated with the most favorable binding conformation. PyMol (Schrödinger, New York, NY, USA) was used for molecular visualization [71].

### 3.4. Microorganisms

As Gram-positive strains, *Staphylococcus aureus* American Type Culture Collection (ATCC) 25923, *Enterococcus faecalis* ATCC 29212 and methicillin and ofloxacin-resistant *Staphylococcus aureus* 272123 clinical isolate were used. As Gram-negative strains, *Escherichia coli* ATCC 25922, *Pseudomonas aeruginosa* ATCC 27853, the *acrA* gene inactivated mutant *Salmonella enterica* serovar Typhimurium SL1344 (SE03), and clinical isolates of the extended-spectrum β-lactamase producer (ESBL) *E. coli* SA/2 were investigated in this study.

The bacteria used for the QS assay were *Chromobacterium violaceum* wild type 85 (wt85), capable of producing violacein, a purple pigment, mediated by AHL signal molecule, capable of endogenous QS-signal molecule production (*N*-hexanoyl-l-HSL), the Tn5 transposase-mutant *C. violaceum* CV026 (CV026), capable of producing violacein in the presence of AHL, but incapable of producing endogenous QS-signal molecules, serving as a detector of external stimuli provided by *Sphingomonas paucimobilis* Ezf 10–17 (EZF), a AHL-producing-strain, and *Serratia marcescens* AS-1, a producer of prodigiosin (2-methyl-3-pentyl-6-methoxyprodigiosin), an orange-red pigment mediated by the AHL-signal molecules, capable of endogenous QS-signal molecule production (*N*-hexanoyl-l-HSL). These bacteria are Gram-negative [60].

As yeast *Candida albicans* ATCC 10231, and as filamentous fungi *Aspergillus fumigatus* ATCC 204305 and a dermatophyte (*Trichophyton rubrum* FF5, clinical isolate) were used. Cultures were obtained in SDA.

### 3.5. Antibacterial Assay and Synergy with Antimicrobials

The antibacterial activity was assessed by determination of the MIC of the compounds using the microdilution method, in a 96-well plate, according to the Clinical and Laboratory Standard Institute (CLSI) guidelines [72]. The media used was MHB II. The concentrations tested ranged from 64 µg/mL to 4 µg/mL, which were later converted to µM. The MIC was determined by visual inspection. DMSO, in subinhibitory concentrations (1% *v/v*), was used as a solvent for the compounds.

The combined effect of the compounds and clinically relevant antimicrobial drugs was evaluated by determining the antibiotic MIC in the presence of each compound. Briefly, the MIC of cefotaxime (CTX) (Duchefa Biochemie, Haarlem, The Netherlands) was determined in the presence of the highest concentration of each compound tested that did not affect bacterial growth of *E. coli* SA/2 when the compound was used alone. The antibiotic tested was serially diluted, whereas the concentration of each compound was kept fixed at 64 µg/mL, which were later converted to µM. Antibiotic MICs were determined as described above.

### 3.6. Antifungal Assay

The susceptibility tests for yeasts were performed based on the Clinical and Laboratory Standards Institute (CLSI) description for the broth microdilution method on the reference document M27A-3 for yeasts [73] and M38-A2 [74] for filamentous fungi, in RPMI-1640 broth culture media. The concentrations tested ranged from 128 µg/mL to 32 µg/mL. The MIC was considered the lowest concentration that was able to totally inhibit the growth when comparing to control (non-treated microorganism in culture medium). A DMSO control (microorganism in RPMI with DMSO (1% *v/v*) was included.

### 3.7. Efflux Pump Inhibition Assay

The efflux pump inhibition assay was carried out using compounds **5–7**, **9**, **10**, **12**, and the enantiomeric pairs **11**, **17**, and **18**, and this effect was studied in the strains SE03 and *S. aureus* 272123. The intracellular accumulation of EB, a known substrate of bacteria efflux pumps, was monitored through real-time fluorimetry. The method used was automated, using a CLARIOstar Plus plate reader (BMG Labtech, Ortenberg, Germany). The positive controls were reserpine and CCCP, and were applied at the non-toxic concentration of 25 µM, as well as DMSO, used as solvent, which was applied at 1% *v/v*. *S. aureus* 272123 and SE03 were incubated in TSB and LB-B, respectively, at 37 °C until they reached an optical density (OD) = 0.4–0.6 at λ = 600 nm. The culture was centrifuged at 13,000× *g* for 3 min, and the pellet was washed and resuspended with PBS. The suspension was centrifuged again in the same conditions and resuspended in PBS. The compounds were diluted to 50 µM in a solution 1 µg/mL of EB, and 50 µL of this solution were transferred into a 96-well black microtiter plate (Greiner Bio-One Hungary Kft, Mosonmagyaróvár, Fertősor, Hungary). Then, 50 µL of bacterial suspension (OD_600_ = 0.4–0.6) were added to each well. The plates were placed into the CLARIOstar plate reader, and the fluorescence was monitored at excitation (530 nm) and emission (600 nm) wavelengths every minute for 1 h on a real-time basis. From the real-time data, the activity of the compounds was expressed as the RFI of the last time point (minute 60) of the EB accumulation assay, which was calculated according to the following formula:

(1) $$\mathrm{RFI}=\;\frac{{\mathrm{RF}}_{\mathrm{treated}}-{\mathrm{RF}}_{\mathrm{untreated}}}{{\mathrm{RF}}_{\mathrm{untreated}}}$$

where RF_treated_ is the relative fluorescence (RF) at the last time point of EB accumulation curve in the presence of the compound, and RF_untreated_ is the RF at the last time point of the EB accumulation curve of the untreated control, having only the solvent (DMSO) control.

### 3.8. Inhibition of Biofilm Formation

The enantiomeric pair composed by compounds **(*S*,*S*)-8** and **(*R*,*R*)-8**, compound **(*R*)-17** and **(*R*,*S*)-18** were tested for their ability to inhibit the formation of biofilm. The bacterial strains used were the Gram-positive *S. aureus* ATCC 25923 and *S. aureus* 272123. The detection of the biofilm formation was possible with the use of the dye crystal violet (CV; 0.1% *v/v*). The initial inoculum was incubated in TSB overnight, and then diluted to an OD_600_ of 0.1. Then, the bacterial suspension was added to 96-well microtiter plates and the compounds were added at a concentration of ½ MIC, and for compounds whose MIC was higher than 100 µM, a concentration of 100 µM was used. The final volume in each well was 200 µL. Reserpine was used as the positive control, as it was the same compound used in the efflux pump inhibition assay and it has shown activity in the inhibition of biofilm formation in *S. aureus* strains [58]. The plates were incubated at 30 °C for 48 h, with gentle stirring (100 rpm). After this incubation period, the TSB medium was discarded, and the plates were washed with tap water to remove unattached cells. Afterwards, 200 µL of a 0.1% *v/v* CV solution were added to the wells and incubated for 15 min at room temperature. Then, the CV solution was removed from the wells, and the plates were washed again with tap water, and 200 µL of a 70% ethanolic solution were added to the wells. The biofilm formation was determined by measuring the OD_600_ using a Multiscan EX ELISA plate reader (Thermo Labsystems, Cheshire, WA, USA). The anti-biofilm effect of the compounds was expressed as the percentage (%) of a decrease in biofilm formation.

### 3.9. Quorum-Sensing Assay

The QS inhibitory effect of the compounds was examined on the EZF and the sensor CV026 strains, on the wt85 strain, and on *S. marcescens*, for **(*S*,*S*)-8**, **(*R*,*R*)-8, (*R*)-17**, and **(*R*,*S*)-18**. The method used was the parallel inoculation method, where pair combinations of the used sensor strain CV026 and the EZF, the AHL-producing strain, were inoculated directly onto the LB*-A agar surface in parallel, at an approximate distance of 5 mm from each other. *S. marcescens* AS-1 and wt85 were inoculated as a single line. Filter paper disks (7 mm in diameter) were placed on the center of the inoculated line(s) and impregnated with 8 µL of a solution of 10 mM of the compounds. PMZ, previously described as a QS inhibitor, was used as the positive control [59]. The agar plates were incubated at room temperature (20 °C) for 24–48 h. The QS inhibition was accessed visually through the inhibition of pigment production. The discolored but intact bacterial colonies were measured with a ruler [59,60,75].

## 4. Conclusions

In this study, a small library of chiral derivatives of xanthones was synthesized, having as chemical model molecules of antimicrobial peptides. Herein, not only the potential of these derivatives as antimicrobial agents were studied but also their usefulness as antimicrobial helpers, capable of acting in resistance mechanisms and possibly as combination therapy with antimicrobials.

Concerning the antimicrobial activity, none of the derivatives tested displayed activity against the bacterial and fungal strains tested. However, when it comes to synergy with antibiotics, it was observed that compounds **6** and **(*S*)-11** were able to decrease the MIC of a β-lactam antibiotic in an ESBL-producing strain of *E. coli*, suggesting the potential of these compounds to also evade resistance mechanisms. It should also be highlighted that, although the correspondent enantiomer was also tested, they were not active in the same way, which leads to the conclusion that enantioselectivity plays an important role in these compounds.

Some chiral derivatives and their precursors were also tested for their potential as inhibitors of efflux pumps. Herein, it was shown that one compound, **(*R*,*R*)-8**, was active as an inhibitor of efflux pumps in the Gram-positive strain tested, whereas three compounds, **(*S*,*S*)-8**, **(*R*)-17**, and **(*R*,*S*)-18** had the same effect in the Gram-negative strain tested. Once again, there was enantioselectivity for this activity in different strains. Moreover, both enantiomers of **8** could be investigated as antimicrobial adjuvants in infections where Gram-positive and Gram-negative multidrug-resistant bacteria were present.

The compounds were also investigated for their ability to inhibit mechanisms related to efflux pumps, such as biofilm formation and QS. It was shown that none of the compounds presented higher inhibition of biofilm formation than reserpine, used as a positive control, but compound **(*R*,*R*)-8** showed the highest inhibition, which may suggest a link between inhibition of efflux pumps and biofilm formation, in this case. The enantiomeric pair composed by compounds **(*S*,*S*)-8** and **(*R*,*R*)-8** also proved successful in inhibiting QS in the tested strains. These results suggest that, in the case of QS, enantioselectivity does not play an important role, rather than the nature of the substituents.

In conclusion, it can be observed that these compounds show potential for the activities tested. Future studies will focus on the evaluation of the cytotoxicity of the compounds in eukaryotic cells and in deeper studies of their mechanisms of efflux pump inhibition. Docking studies presented suggest that these compounds can interact with the most relevant efflux pumps, but other mechanisms, such as membrane disturbance, which is characteristic of antimicrobial peptides, could also play a role in the obtained results.

## Figures and Tables

**Table 1:** Docking results for the studied compounds in bacterial efflux pumps.

Compound	Docking Score						
	AcrA		AcrB		TolC	NorA	
	HH	LD	SBS	HT		BCR	CS
**5**	−6.1	−6.8	−7.5	−4.9	−6.5	−5.8	−5.3
**6**	−5.7	−5.6	−7.3	−5.3	−6.5	−6.5	−6.4
**7**	−6.0	−5.9	−8.0	−4.4	−7.0	−6.1	−5.5
**(*S*,*S*)-8**	−7.7	−6.3	−7.6	−5.2	−7.1	−7.7	−5.8
**(*R*,*R*)-8**	−6.9	−5.8	−8.3	−5.2	−7.1	−5.9	−5.0
**9**	−5.9	−5.8	−7.3	−5.2	−6.5	−6.1	−5.8
**10**	−5.8	−6.2	−7.3	−5.1	−6.7	−6.2	−5.8
**(*S*)-11**	−7.4	−5.4	−7.6	1.6	−7.9	−8.1	−5.6
**(*R*)-11**	−6.7	−4.8	−7.3	−6.1	−7.8	−7.8	−5.6
**12**	−5.7	−5.7	−6.9	−5.6	−6.7	−5.4	−5.3
**(*S*)-16**	−6.8	−6.0	−8.9	−6.9	−7.5	−7.2	−6.4
**(*R*)-16**	−6.6	−6.4	−9.1	−6.7	−7.8	−5.2	−6.0
**(*S*)-17**	−7.3	−6.2	−9.2	3.2	−7.9	−7.0	−5.2
**(*R*)-17**	−7.0	−5.5	−8.5	0.0	−8.1	−8.7	−5.1
**(*S,R*)-18**	−7.5	−6.9	−9.6	−7.2	−9.0	−5.9	−6.5
**(*R,S*)-18**	−7.3	−5.8	−9.8	−7.1	−8.4	-7.1	-5.7
**Reserpine**	5.6	4.6	−8.7	10.9	−7.5	1.0	−4.6
**PAβN**	−5.8	−4.9	−7.1	−4.7	−7.1	−9.4	−5.3
**D13-9001**	−6.2	−5.1	−9.7	26.5	−7.4	ND	ND
**Doxorubicin**	−7.2	−5.6	−8.9	15.4	−7.2	ND	ND
**MBX-3132**	−7.9	−6.2	−7.9	2.9	−7.7	ND	ND
**Minocycline**	−6.2	−5.4	−8.7	26.7	−7.7	ND	ND

**SBS**: Substrate-binding site; **HT**: Hydrophobic trap; **HH**: Helical hairpin; **LD**: Lipoyl domain; **BCR**: Binding core region; **CS**: Cytoplasmic side; **ND**: Not determined.

**Table 2:** Relative fluorescence index (RFI) of the tested chiral xanthones and precursors.

Compound	RFI ± SD	
	*S. aureus* 272123	SE03
**5**	0.11 ± 0.05	0.10 ± 0.01
**6**	0.01 ± 0.04	0.05 ± 0.07
**7**	−0.14 ± 0.01	−0.06 ± 0.04
**(*S*,*S*)-8**	−0.21 ± 0.01	2.23 ± 0.05
**(*R*,*R*)-8**	3.19 ± 0.31	0.03 ± 0.06
**9**	0.03 ± 0.03	0.36 ± 0.05
**10**	−0.01 ± 0.01	−0.03 ± 0.02
**(*S*)-11**	−0.22 ± 0.07	0.05 ± 0.07
**(*R*)-11**	0.15 ± 0.12	−0.03 ± 0.07
**12**	0.14 ± 0.03	0.28 ± 0.02
**(*S*)-17**	0.05 ± 0.04	0.13 ± 0.05
**(*R*)-17**	0.30 ± 0.02	4.64 ± 0.23
**(*S,R*)-18**	0.05 ± 0.01	0.02 ± 0.06
**(*R,S*)-18**	0.26 ± 0.07	0.96 ± 0.04
**Reserpine**	0.42 ± 0.13	ND
**CCCP**	ND	0.44 ± 0.03

**SE03**: *S. enterica* serovar Typhimurium SL1344; **SD**: Standard deviation; **CCCP**: Carbonyl cyanide 3-chlorophenylhydrazone; **ND**: Not determined.

**Table 3:** Biofilm and quorum sensing inhibition of the chiral xanthones tested.

Compound	Biofilm Inhibition (%) ± SD		Quorum Sensing Inhibition (mm) ± SD		
	*S. aureus* ATCC 29213	*S. aureus* 272123	*S. marcescens*	wt85	EZF + CV026
**(*S*,*S*)-8**	0	7.85 ± 1.95	0	0	47 ± 0.1
**(*R*,*R*)-8**	0	55.59 ± 3.26	0	0	31 ± 0.8
**(*R*)-17**	0	46.23 ± 1.19	0	0	0
**(*R,S*)-18**	0	21.04 ± 1.47	0	0	0
**Reserpine**	22.29 ± 5.10	63.1 ± 2.33	ND	ND	ND
**PMZ**	ND	ND	18 ± 0.8	40 ± 0.1	41 ± 0.5

**wt85**: *Chromobacterium violaceum* wild-type 85; **EZF**: *Sphingomonas paucimobilis* Ezf 10–17; **CV026**: *C. violaceum* CV026; **SD**: Standard deviation; **PMZ**: Promethazine; **ND**: not determined.

## Data Availability

Not applicable.

## Supplementary Materials

The following are available online at https://www.mdpi.com/article/10.3390/ph14111141/s1, Table S1: Minimum inhibitory concentrations of the compounds in the antibacterial and antifungal activity assays and synergy with antibiotics; Figure S1. ^1^H NMR (300.13 MHz, DMSO-*d*6) and ^13^C NMR (75.48 MHz, DMSO-*d*6) for compound **9**; Figure S2. Electrospray ESI data for compound **9**; Figure S3. ^1^H NMR (300.13 MHz, DMSO-*d*6) and ^13^C NMR (75.48 MHz, DMSO-*d*6) for compound **10**; Figure S4. Electrospray ESI data for compound **10**; Figure S5. Chromatograms for the enantioseparation of the enantiomeric pair **11**, at optimized chromatographic conditions; Figure S6. ^1^H NMR (300.13 MHz, DMSO-*d*6) and ^13^C NMR (75.48 MHz, DMSO-*d*6) for the enantiomeric pair **11**; Figure S7. Electrospray ESI data for enantiomeric pair **11**; Figure S8. ^1^H NMR (300.13 MHz, CDCl_3_) and ^13^C NMR (75.48 MHz, DMSO-*d*6) for compound **12**; Figure S9. Electrospray ESI data for compound **12**; Figure S10. ^1^H NMR (300.13 MHz, DMSO-*d*6) and ^13^C NMR (75.48 MHz, DMSO-*d*6) for the enantiomeric pair **16**; Figure S11. Electrospray ESI data for enantiomeric pair **16**; Figure S12. Chromatograms for the enantioseparation of the enantiomeric pair **16**, at optimized chromatographic conditions; Figure S13. ^1^H NMR (300.13 MHz, DMSO-*d*6) and ^13^C NMR (75.48 MHz, DMSO-*d*6) for the enantiomeric pair **17**; Figure S14. Electrospray ESI data for enantiomeric pair **17**; Figure S15. Chromatograms for the enantioseparation of the enantiomeric pair **18**, at optimized chromatographic conditions; Figure S16. ^1^H NMR (300.13 MHz, DMSO-*d*6) and ^13^C NMR (75.48 MHz, DMSO-*d*6) for the enantiomeric pair **18**; Figure S17. Electrospray ESI data for enantiomeric pair **18**.
