# Supplementary material for: Enantioselectivity of Chiral Derivatives of Xanthones in Virulence Effects of Resistant Bacteria
Source: Pharmaceuticals (Basel). 2021 Nov 10;14(11):1141. doi: 10.3390/ph14111141 (PMC8623869; doi:10.3390/ph14111141)
Supplement: Supplementary file 1 [file pharmaceuticals-14-01141-s001.zip › pharmaceuticals-1450525-supplementary.pdf]

Supplementary Data

Enantioselectivity of Chiral Derivatives of Xanthones in Virulence Effects of Resistant Bacteria

Fernando Durães [1,2], Sara Cravo [1,2], Joana Freitas-Silva [2,3], Nikoletta Szemerédi [4], Paulo Martins-da-Costa [2,3], Eugénia Pinto [2,5], Maria Elizabeth Tiritan [1,2,6], Gabriella Spengler [4], Carla Fernandes [1,2]*, Emília Sousa [1,2]*, Madalena Pinto [1,2]

1 Laboratory of Organic and Pharmaceutical Chemistry, Department of Chemical Sciences, Faculty of Pharmacy, University of Porto, Rua de Jorge Viterbo Ferreira, 228, 4050-313 Porto, Portugal; fduraes5@gmail.com (F.D.); scravo@ff.up.pt (S.C.); madalena@ff.up.pt (M.P.)

2 CIIMAR – Interdisciplinary Centre of Marine and Environmental Research, University of Porto, Novo Edifício do Terminal de Cruzeiros do Porto de Leixões, Avenida General Norton de Matos, S/N, 4450-208 Matosinhos, Portugal; joanafreitasdasilva@gmail.com (J.F.-S.); pmcosta@icbas.up.pt (P.M.-d.-C.)

3 ICBAS – Institute of Biomedical Sciences Abel Salazar, Universidade do Porto, Rua de Jorge Viterbo Ferreira 228, 4050-313 Porto, Portugal

4 Department of Medical Microbiology, Albert Szent-Györgyi Health Center and Faculty of Medicine, University of Szeged, Semmelweis utca 6, 6725 Szeged, Hungary; szemeredi.nikoletta@med.u-szeged.hu (N.S.); spengler.gabriella@med.u-szeged.hu (G.S.)

5 Laboratory of Microbiology, Department of Biological Sciences, Faculty of Pharmacy, University of Porto, Rua de Jorge Viterbo Ferreira, 228, 4050-313 Porto, Portugal; epinto@ff.up.pt (E.P.)

6 CESPU, Institute of Research and Advanced Training in Health Sciences and Technologies (IINFACTS), Rua Central de Gandra, 1317, 4585-116 Gandra, Portugal; elizabeth.tiritan@iucs.cespu.pt (M.E.T.)

* Correspondence: cfernandes@ff.up.pt (C.F.); esousa@ff.up.pt (E.S.)

Table S1. Minimum inhibitory concentrations of the compounds in the antibacterial and antifungal activity assays and synergy with antibiotics.

Compound	Antibacterial activity						Synergy with antimicrobials		Antifungal activity		
	Minimum Inhibitory Concentration (MIC) (μM)										
							E. coli SA/2	*E. faecalis* B3/101			
	E. coli ATCC 25922	*P. aeruginosa* ATCC 27853	*E. faecalis* ATCC 29212	*S. aureus* ATCC 29213	*S. aureus* 272123	SE03	CTX MIC = 562 (256 μg/mL)	VAN MIC = 707 (1024 μg/mL)	*C. albicans* ATCC 10231	*A. fumigatus* ATCC 204305	*T. rubrum* FF5
							CTX + Compound[1]	VAN + Compound[1]			
**5**	>100	>100	>100	>100	>100	>100	562 (256 μg/mL)	707 (1024 μg/mL)	>100	>100	>100
**6**	>100	>100	>100	>100	>100	>100	141 (64 μg/mL)	707 (1024 μg/mL)	>100	>100	>100
**7**	>100	>100	>100	>100	>100	>100	562 (256 μg/mL)	707 (1024 μg/mL)	>100	>100	>100
**(*S*,*S*)-8**	>100	>100	>100	>100	>100	>100	562 (256 μg/mL)	707 (1024 μg/mL)	>100	>100	>100
**(*R*,*R*)-8**	>100	>100	>100	>100	>100	>100	562 (256 μg/mL)	707 (1024 μg/mL)	>100	>100	>100
**9**	>100	>100	>100	>100	>100	>100	562 (256 μg/mL)	707 (1024 μg/mL)	>100	>100	>100
**10**	>100	>100	>100	>100	>100	>100	562 (256 μg/mL)	707 (1024 μg/mL)	>100	>100	>100
**(*S*)-11**	>100	>100	>100	>100	>100	>100	141 (64 μg/mL)	707 (1024 μg/mL)	>100	>100	>100
**(*R*)-11**	>100	>100	>100	>100	>100	>100	562 (256 μg/mL)	707 (1024 μg/mL)	>100	>100	>100
**12**	>100	>100	>100	>100	>100	>100	562	707	>100	>100	>100

							(256 μg/mL)	(1024 μg/mL)			
**(*S*)-16**	>100	>100	>100	>100	>100	>100	562 (256 μg/mL)	707 (1024 μg/mL)	>100	>100	>100
**(*R*)-16**	>100	>100	>100	>100	>100	>100	562 (256 μg/mL)	707 (1024 μg/mL)	>100	>100	>100
**(*S*)-17**	>100	>100	>100	>100	>100	>100	562 (256 μg/mL)	707 (1024 μg/mL)	>100	>100	>100
**(*R*)-17**	>100	>100	>100	>100	>100	>100	562 (256 μg/mL)	707 (1024 μg/mL)	>100	>100	>100
**(*S*,*R*)-18**	>100	>100	>100	>100	>100	>100	562 (256 μg/mL)	707 (1024 μg/mL)	>100	>100	>100
**(*R*,*S*)-18**	>100	>100	>100	>100	>100	>100	562 (256 μg/mL)	707 (1024 μg/mL)	>100	>100	>100

[1] All compounds were kept at the highest concentration tested in the antibacterial activity (64 μg/mL), considering that none showed a direct inhibitory effect on *E. coli* and *E. faecalis*. **CTX**: cefotaxime; **VAN**: vancomycin. **SE03**: *Salmonella enterica* serovar Typhimurium SL1344 (SE03).

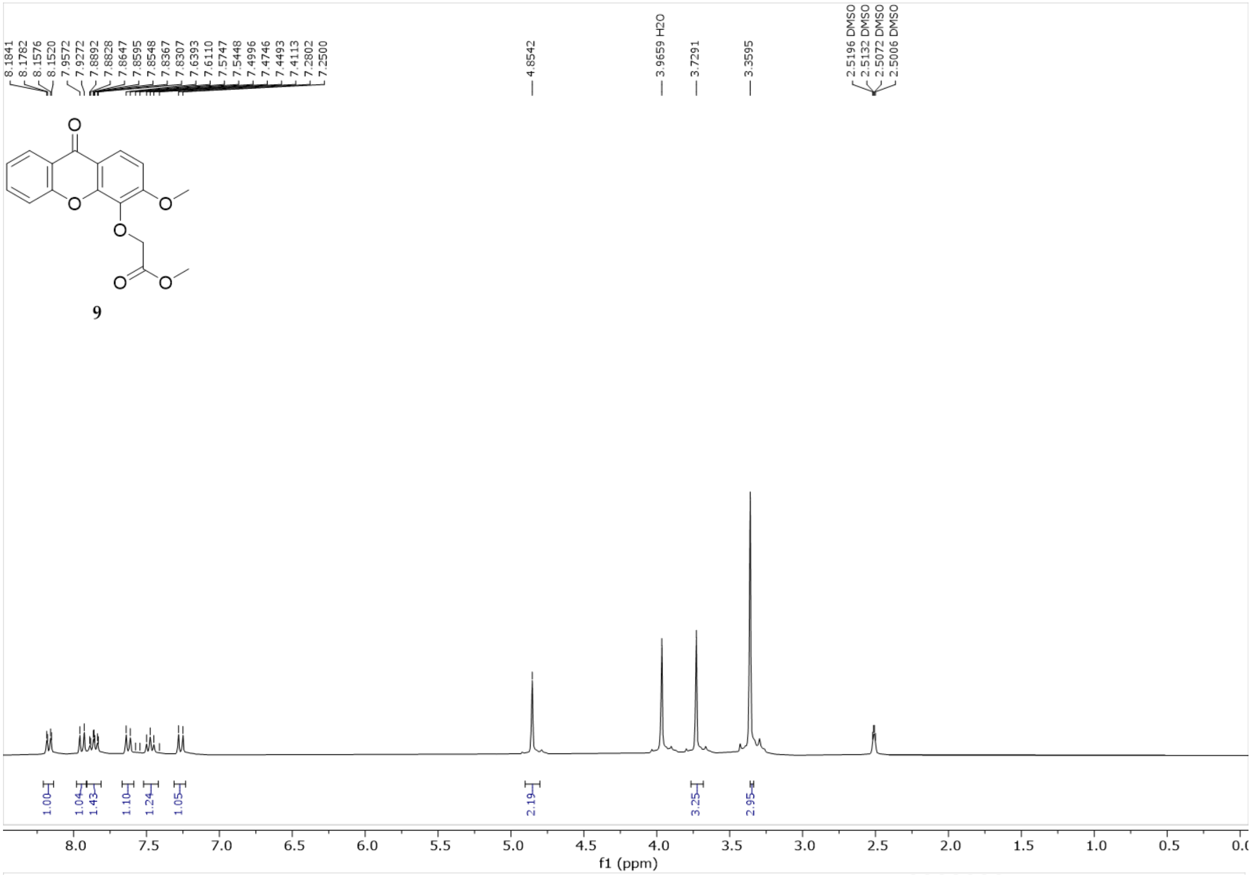

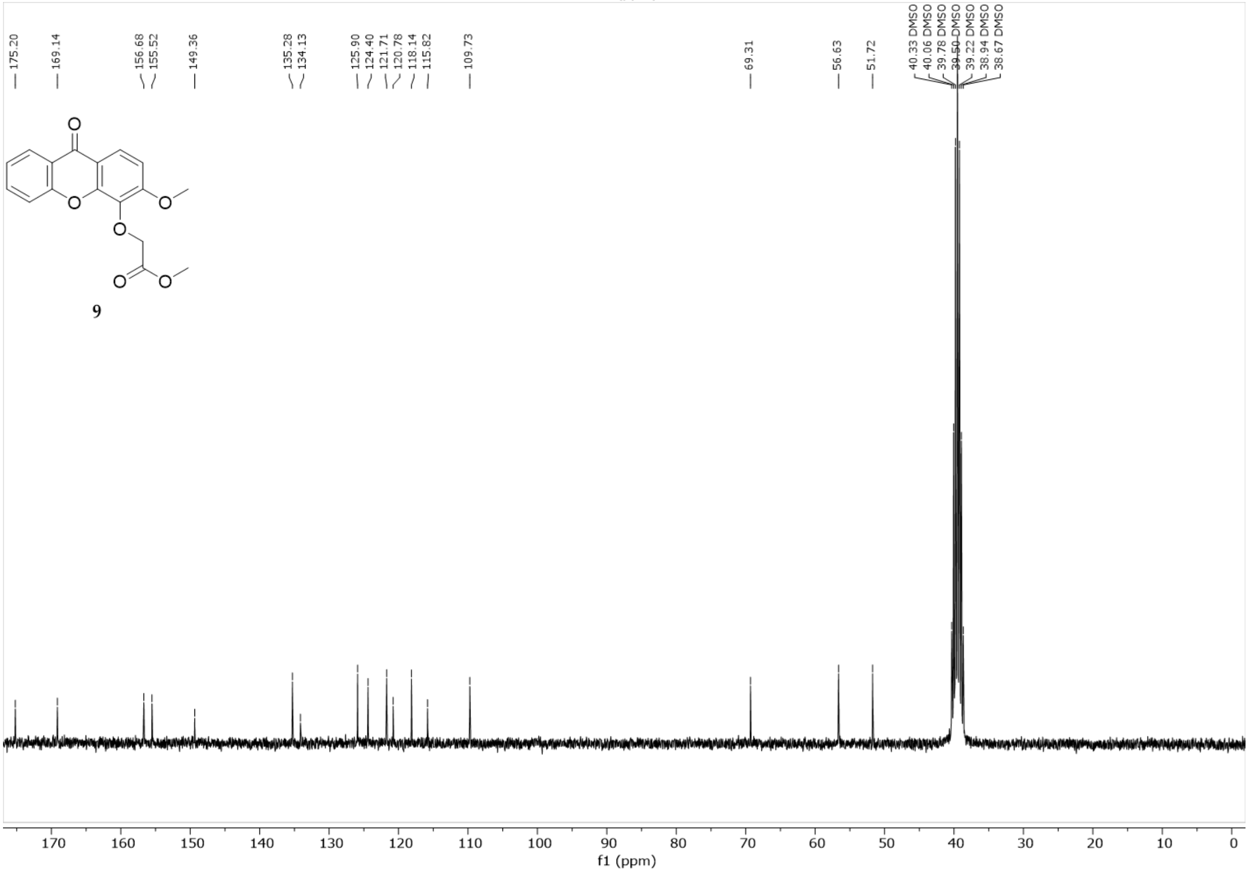

Figure S1. ^{1}H NMR (300.13 MHz, DMSO-*d*6) and ^{13}C NMR (75.48 MHz, DMSO-*d*6) for compound **9**.

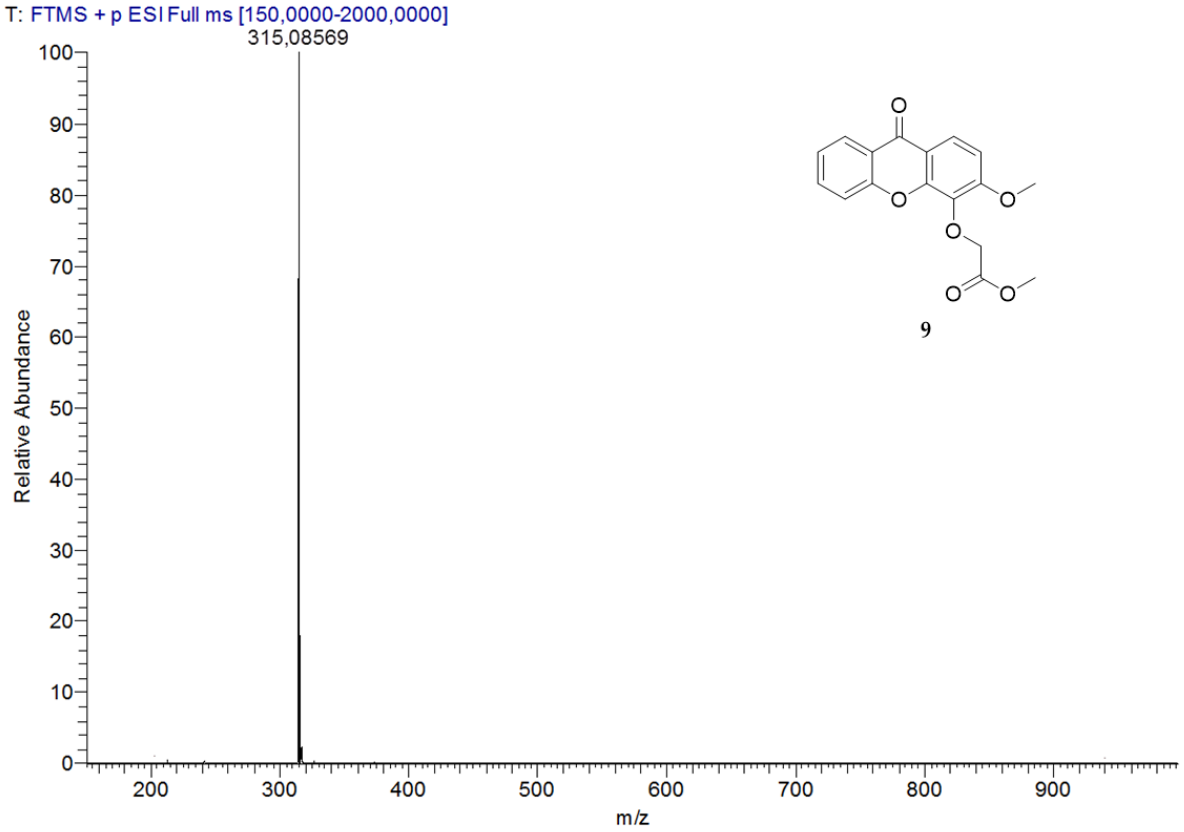

Meas. m/z	Formula	m/z	err [ppm]
314.07904	$C_{17}H_{15}O_6$	315.08569	1.982

Figure S2. Electrospray ESI data for compound **9**.

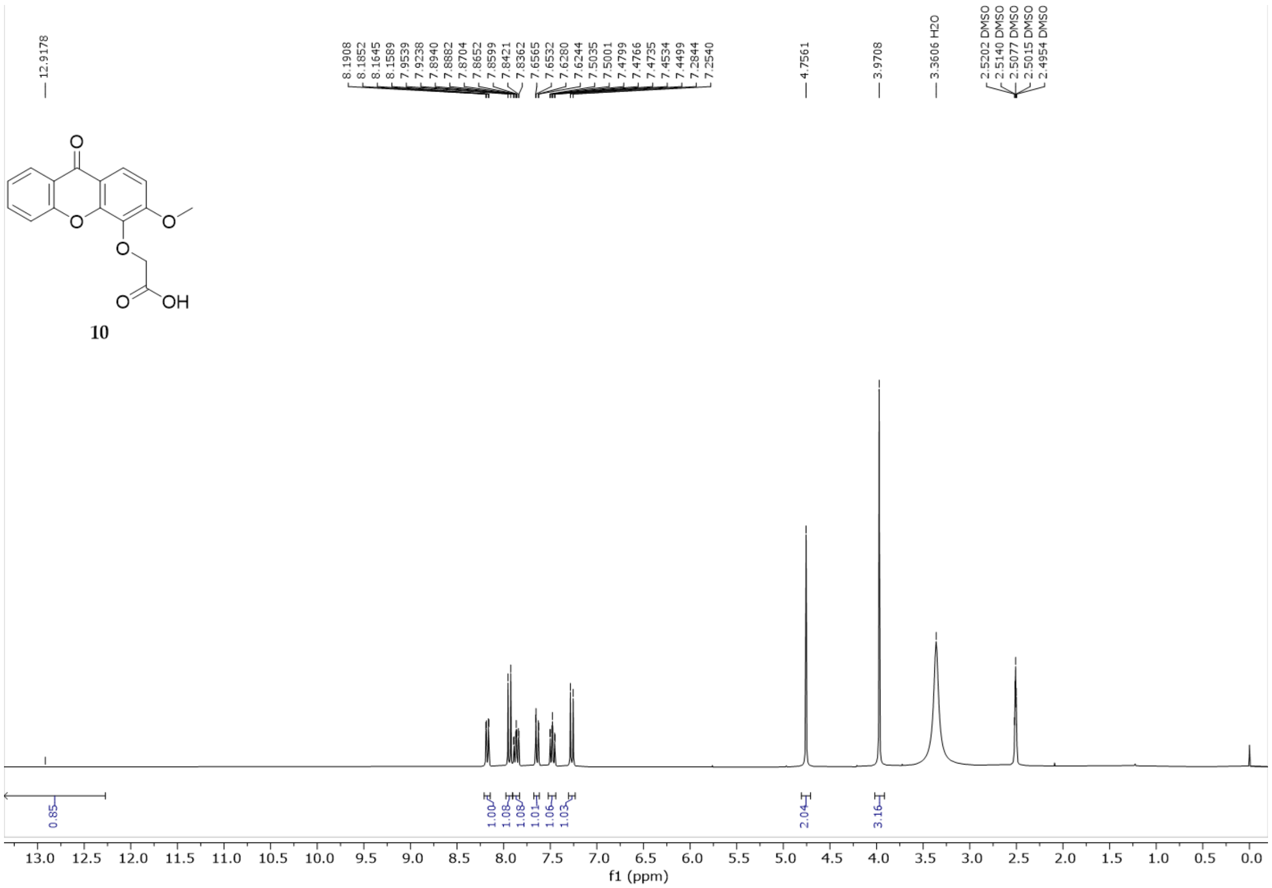

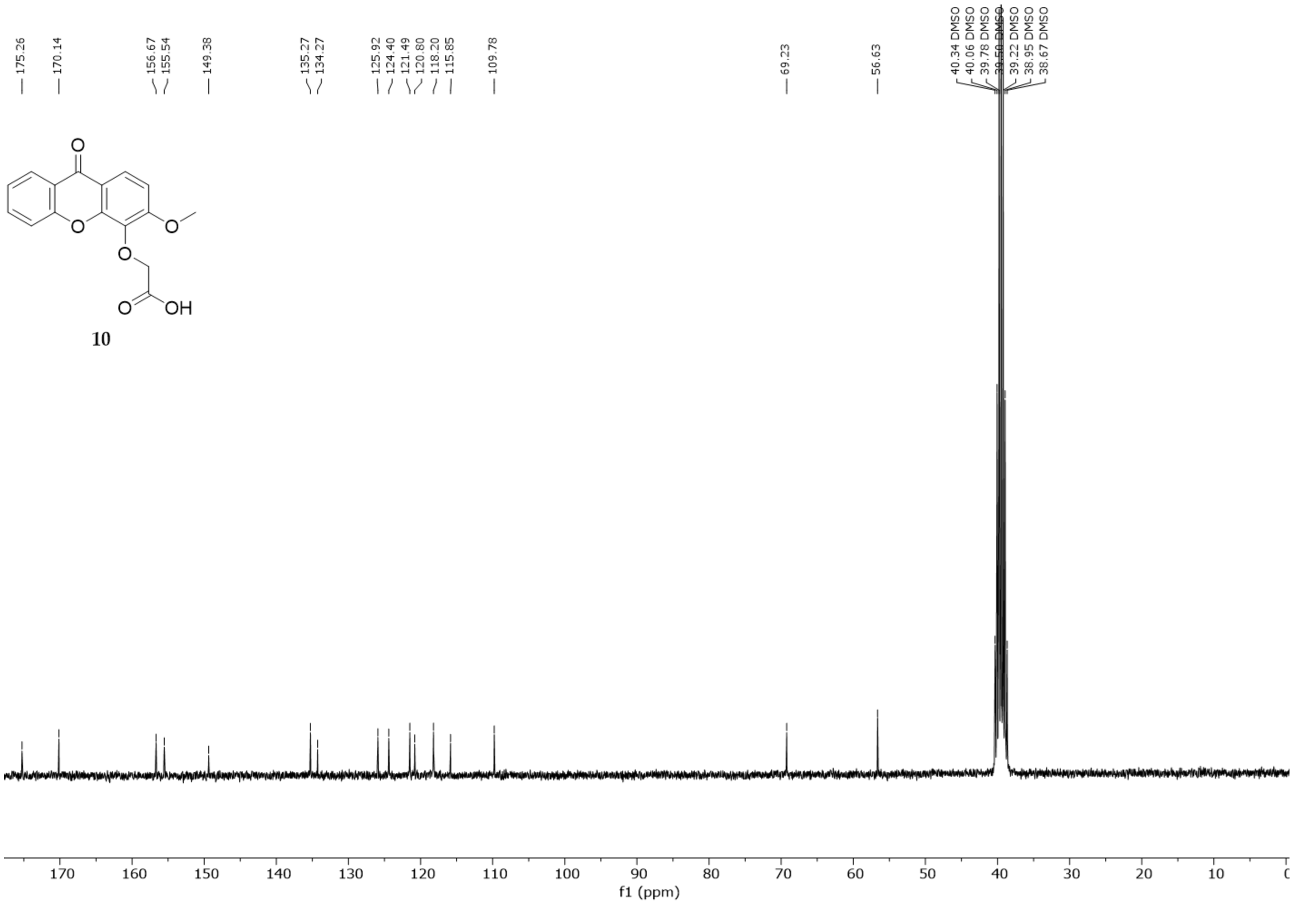

Figure S3. ^{1}H NMR (300.13 MHz, DMSO-*d6*) and ^{13}C NMR (75.48 MHz, DMSO-*d6*) for compound **10**.

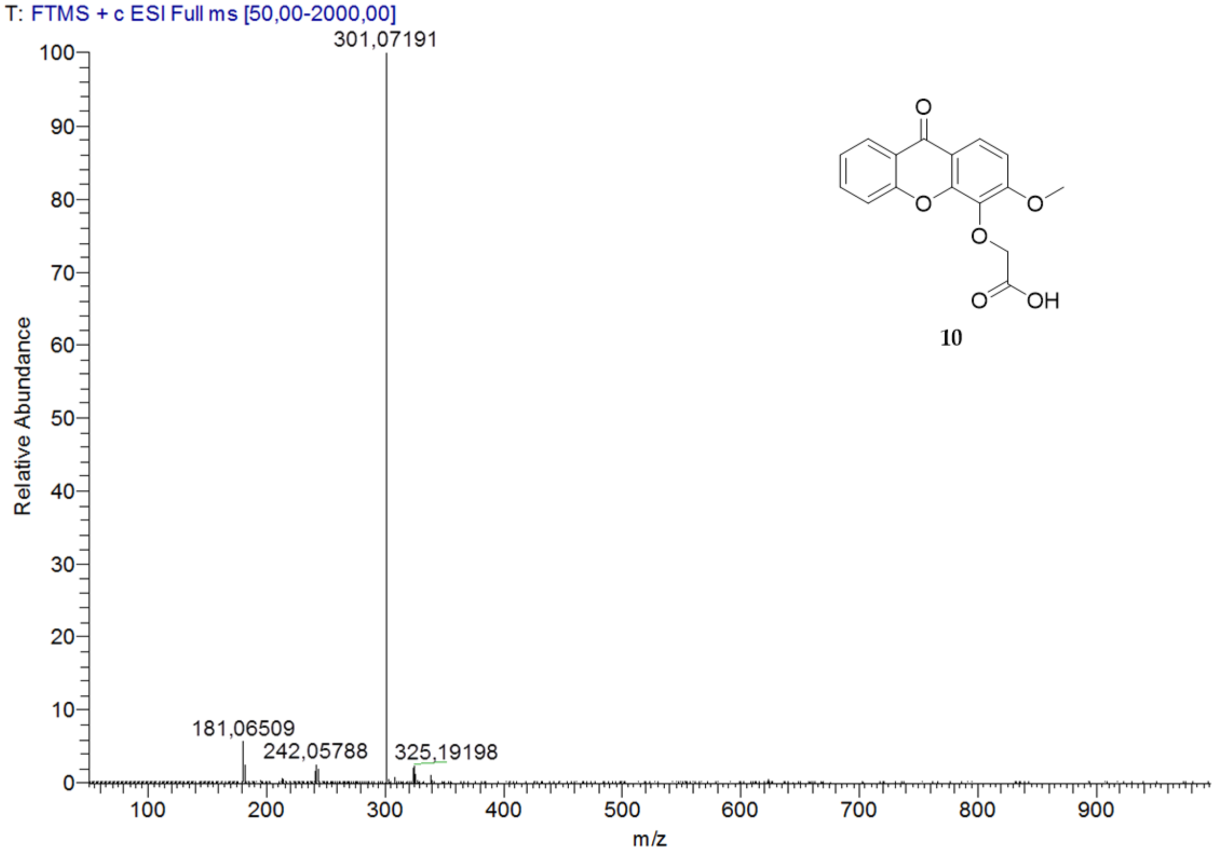

10

Meas. m/z	Formula	m/z	err [ppm]
301.07122	$C_{16}H_{13}O_6$	301.07191	4.137

Figure S4. Electrospray ESI data for compound **10**.

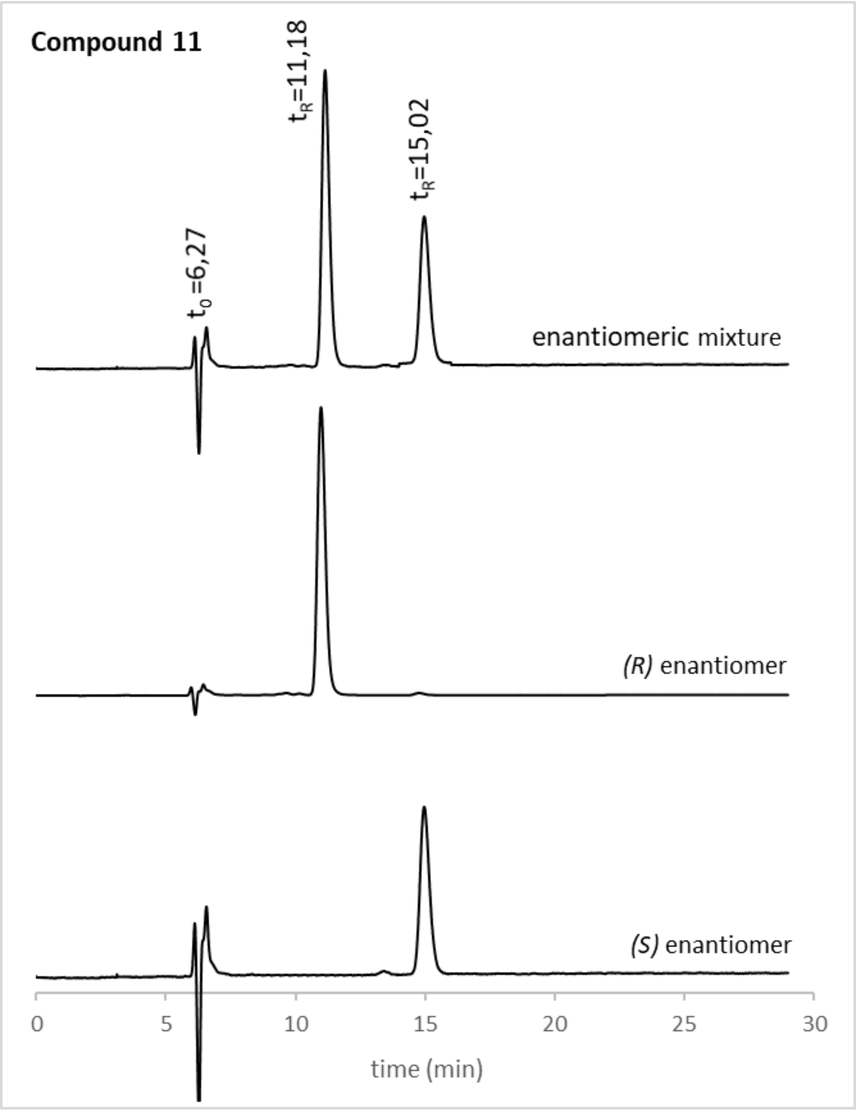

Figure S5. Chromatograms for the enantioseparation of the enantiomeric pair **11**, at optimized chromatographic conditions.

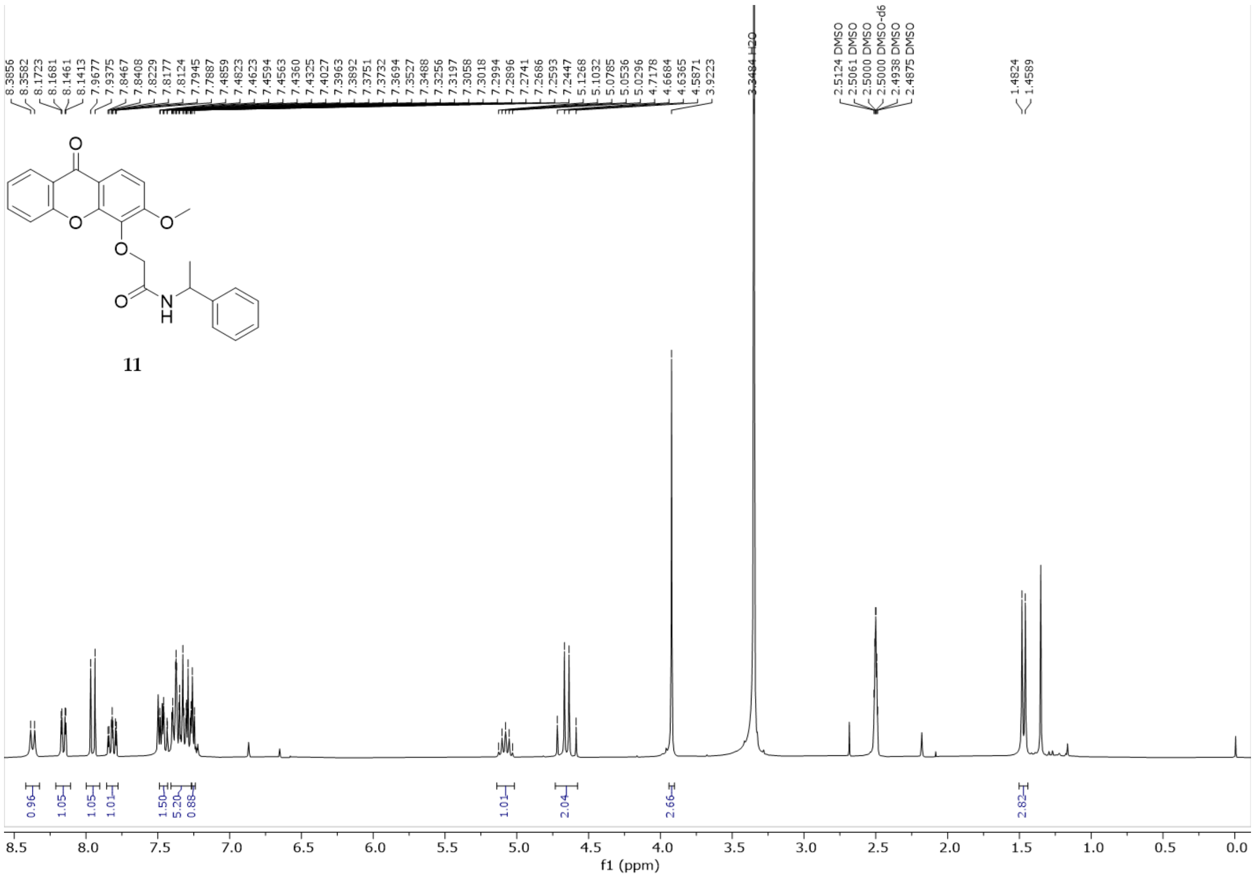

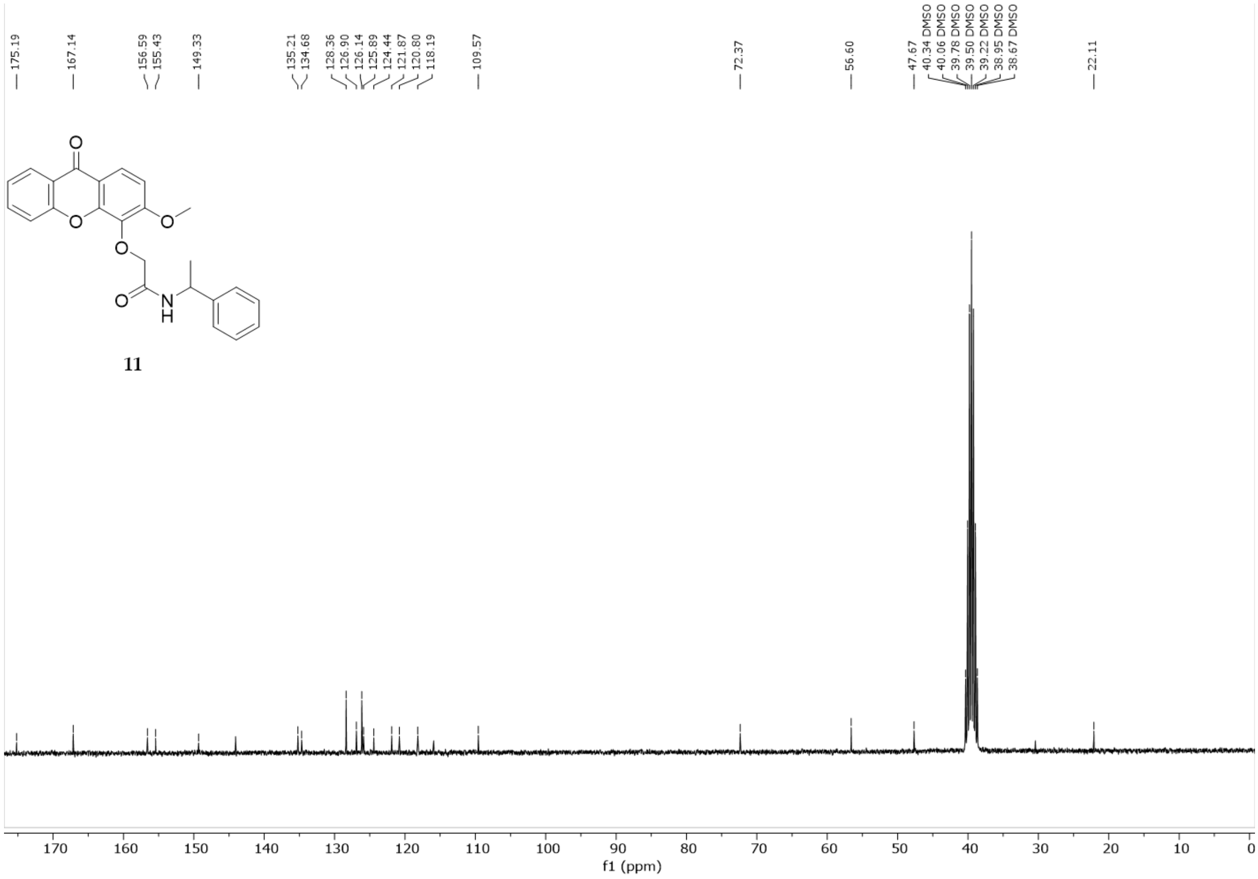

Figure S6. ^{1}H NMR (300.13 MHz, DMSO-*d*6) and ^{13}C NMR (75.48 MHz, DMSO-*d*6) for the enantiomeric pair **11**.

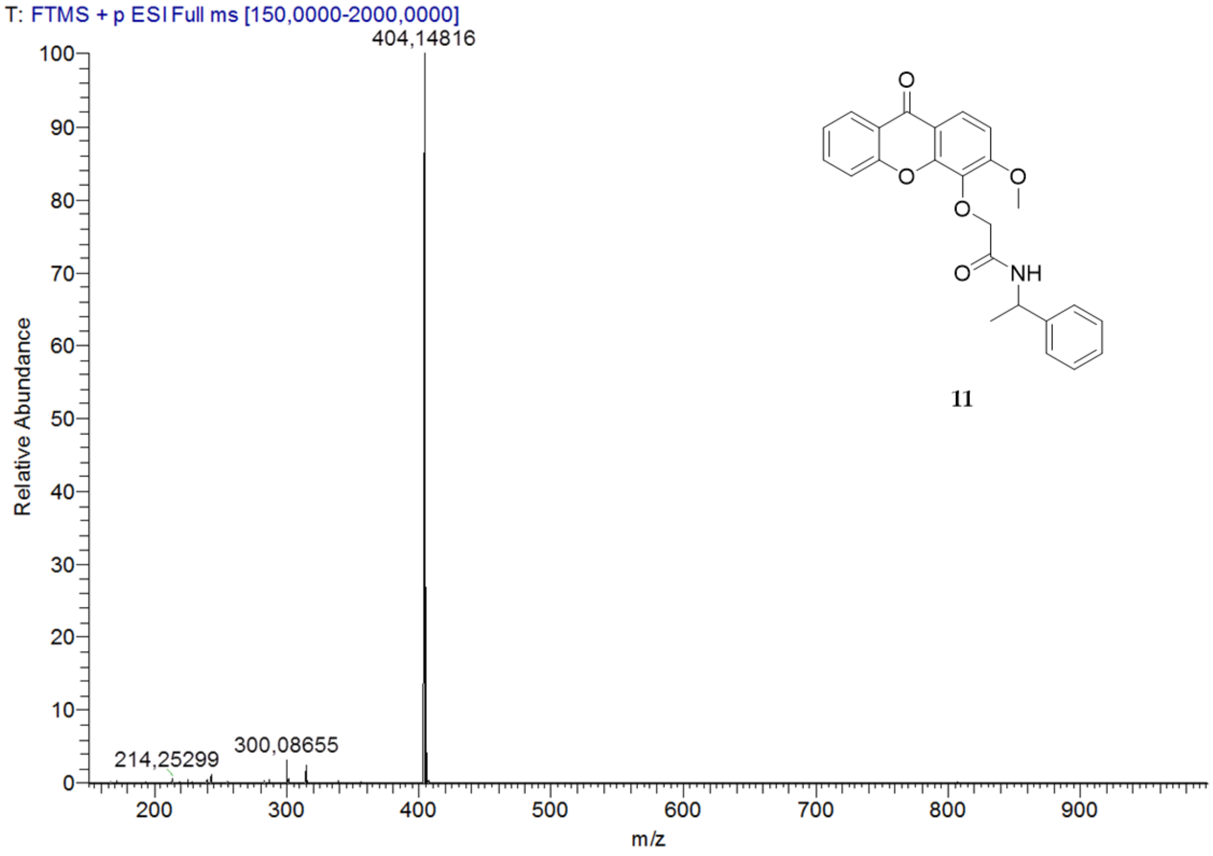

Meas. m/z	Formula	m/z	err [ppm]
404.14980	$C_{24}H_{22}NO_5$	404.14816	2.695

Figure S7. Electrospray ESI data for the enantiomeric pair **11**.

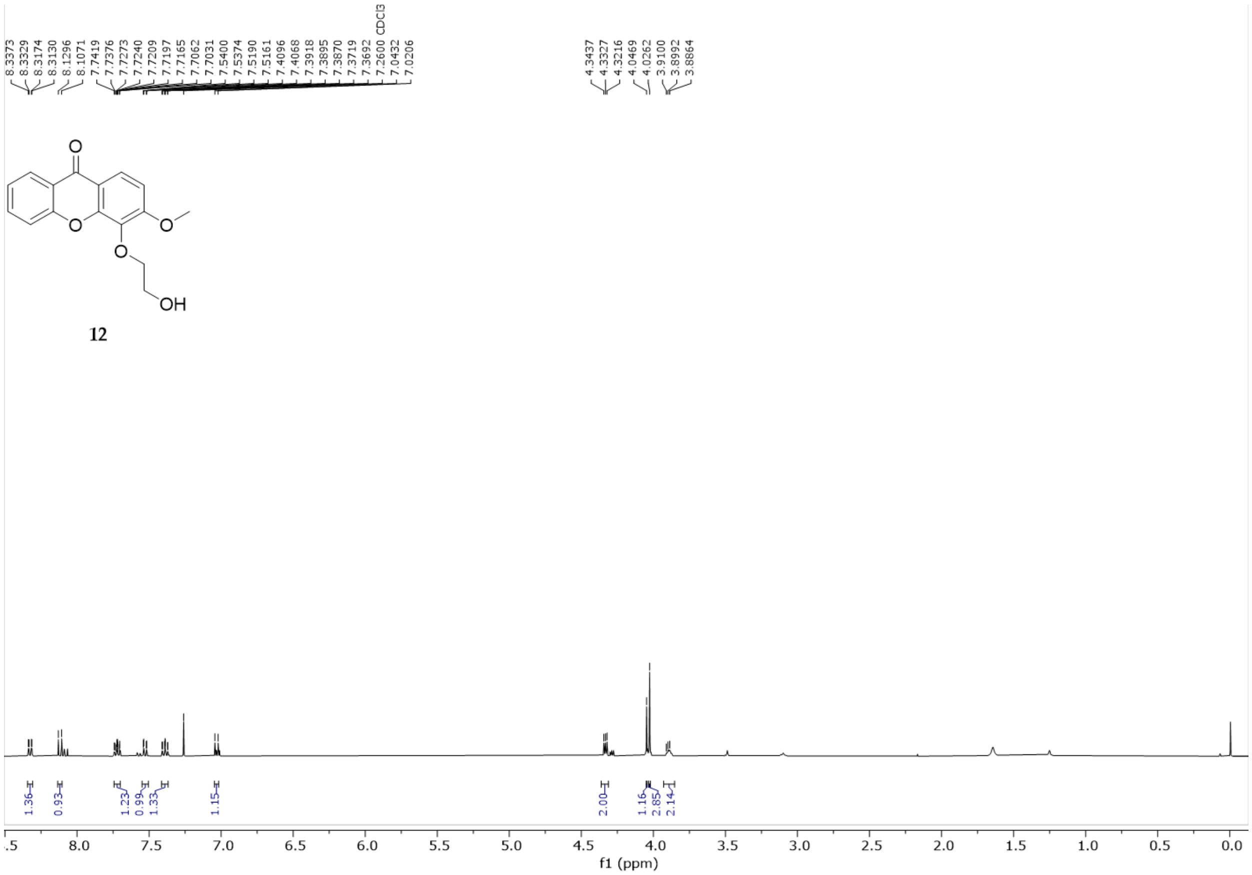

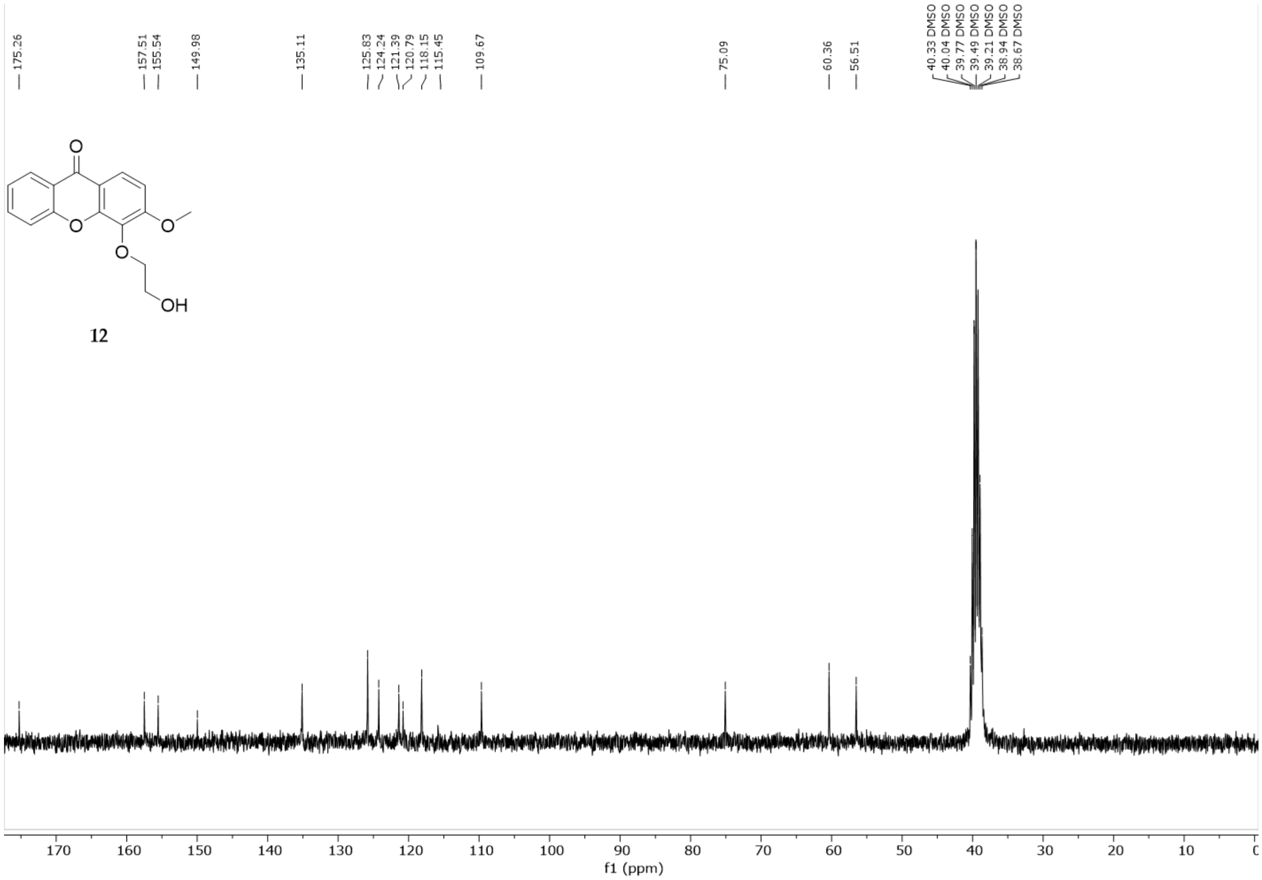

Figure S8. ^{1}H NMR (300.13 MHz, $CDCl_3$) and ^{13}C NMR (75.48 MHz, DMSO-*d*6) for compound **12**.

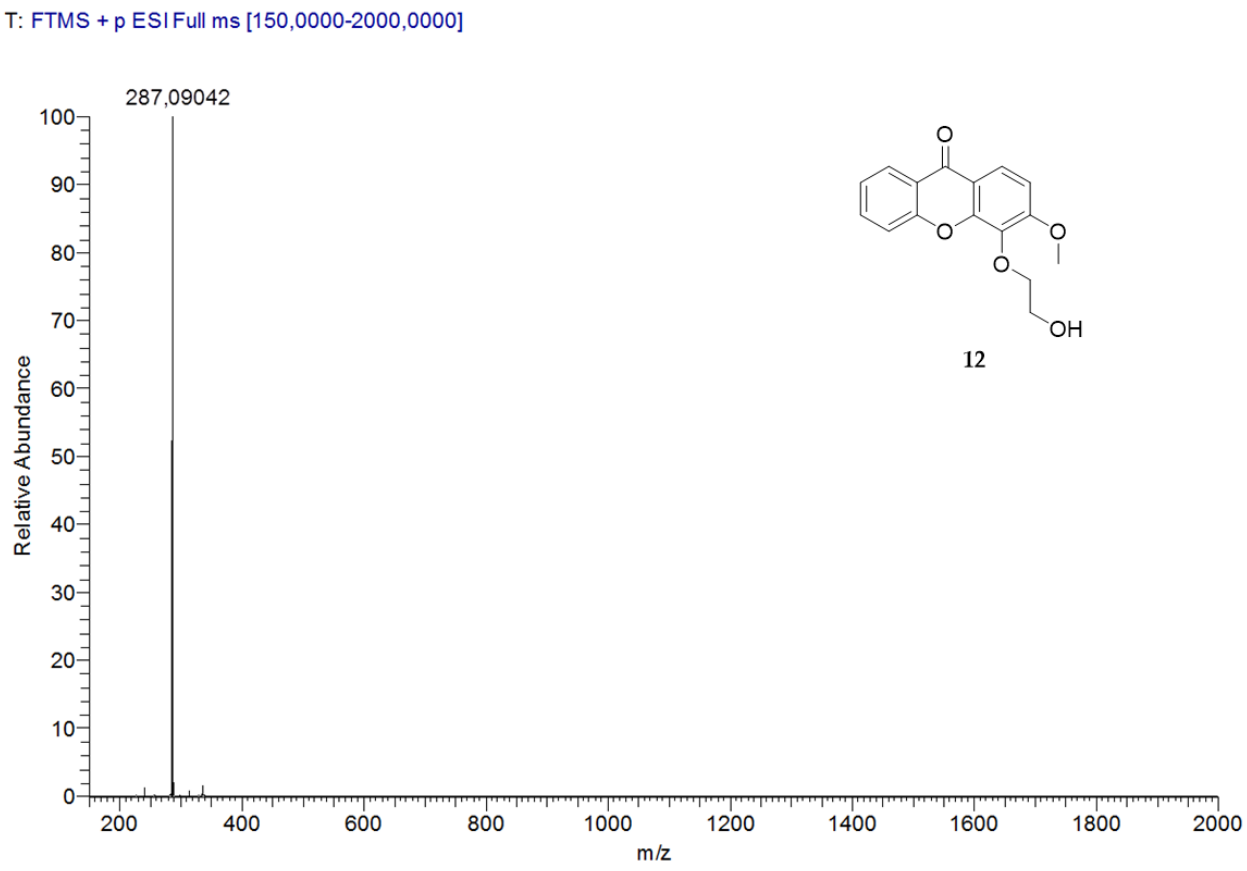

Meas. m/z	Formula	m/z	err [ppm]
287.09195	$C_{16}H_{15}O_5$	287.09042	-3.414

Figure S9. Electrospray ESI data for compound **12**.

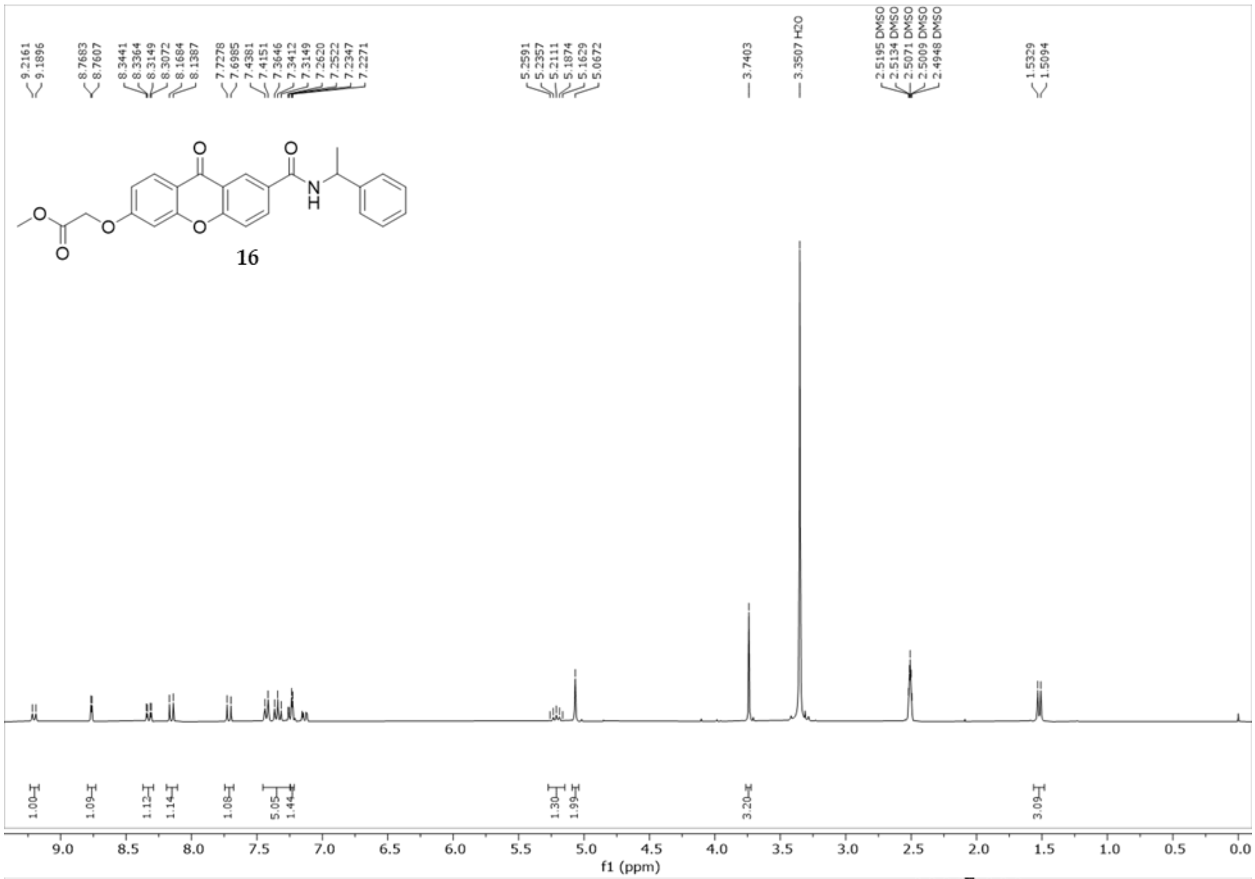

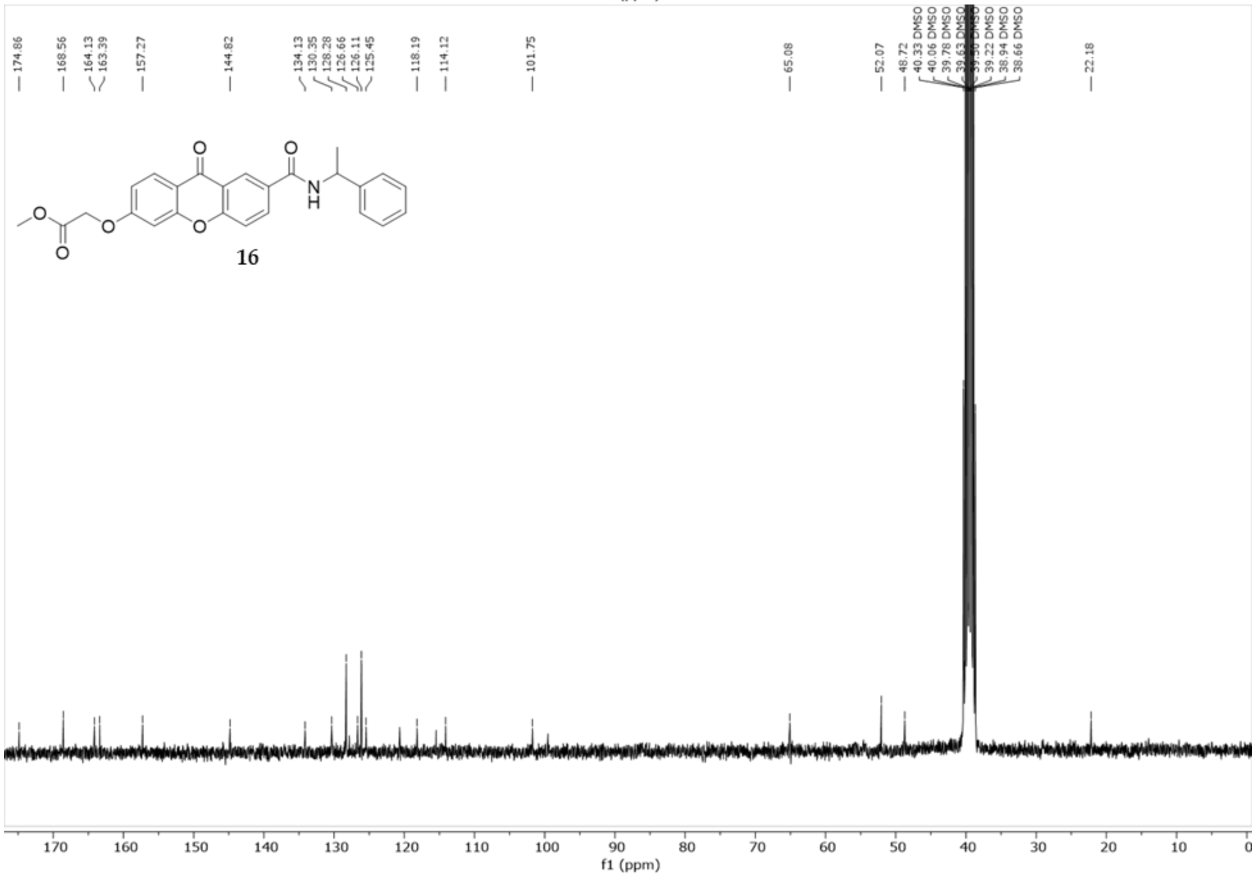

Figure S10. ^{1}H NMR (300.13 MHz, DMSO-*d6*) and ^{13}C NMR (75.48 MHz, DMSO-*d6*) for the enantiomeric pair **16**.

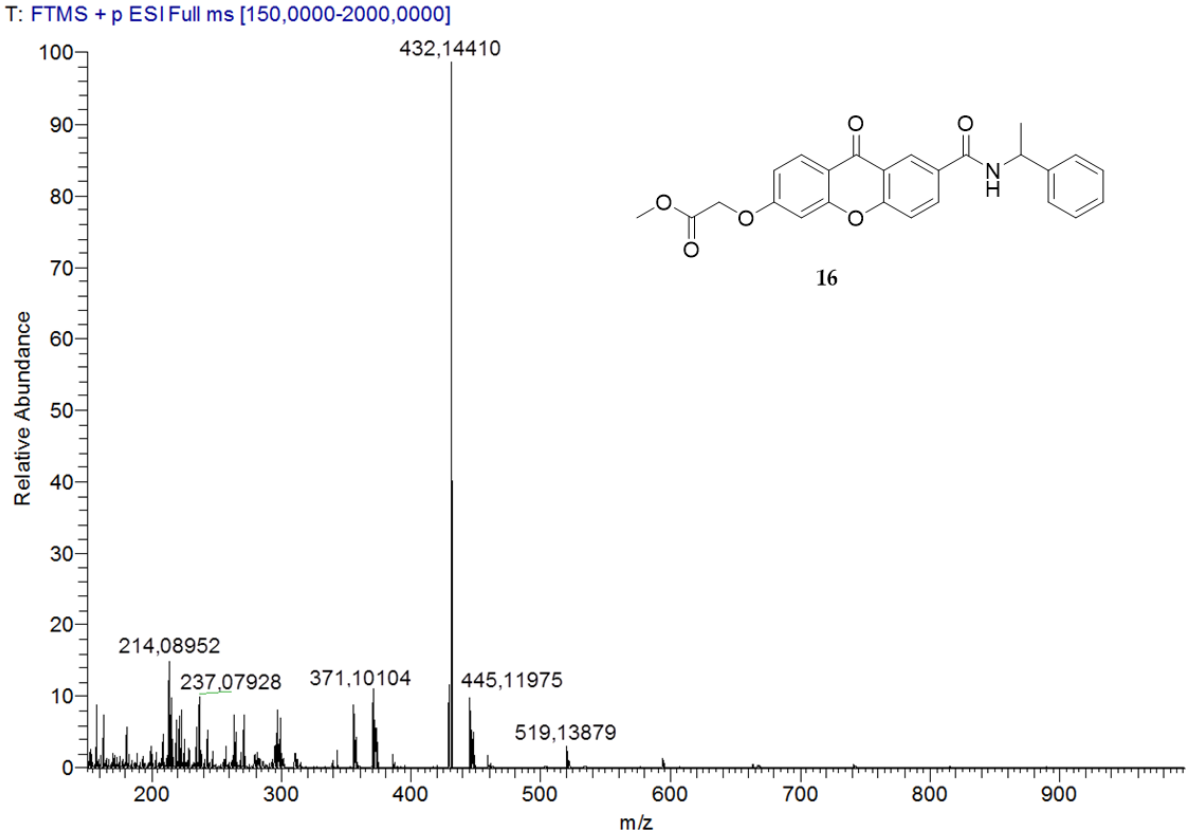

Meas. m/z	Formula	m/z	err [ppm]
432.14471	$C_{25}H_{22}NO_6$	432.14410	-0.148

Figure S11. Electrospray ESI data for the enantiomeric pair **16**.

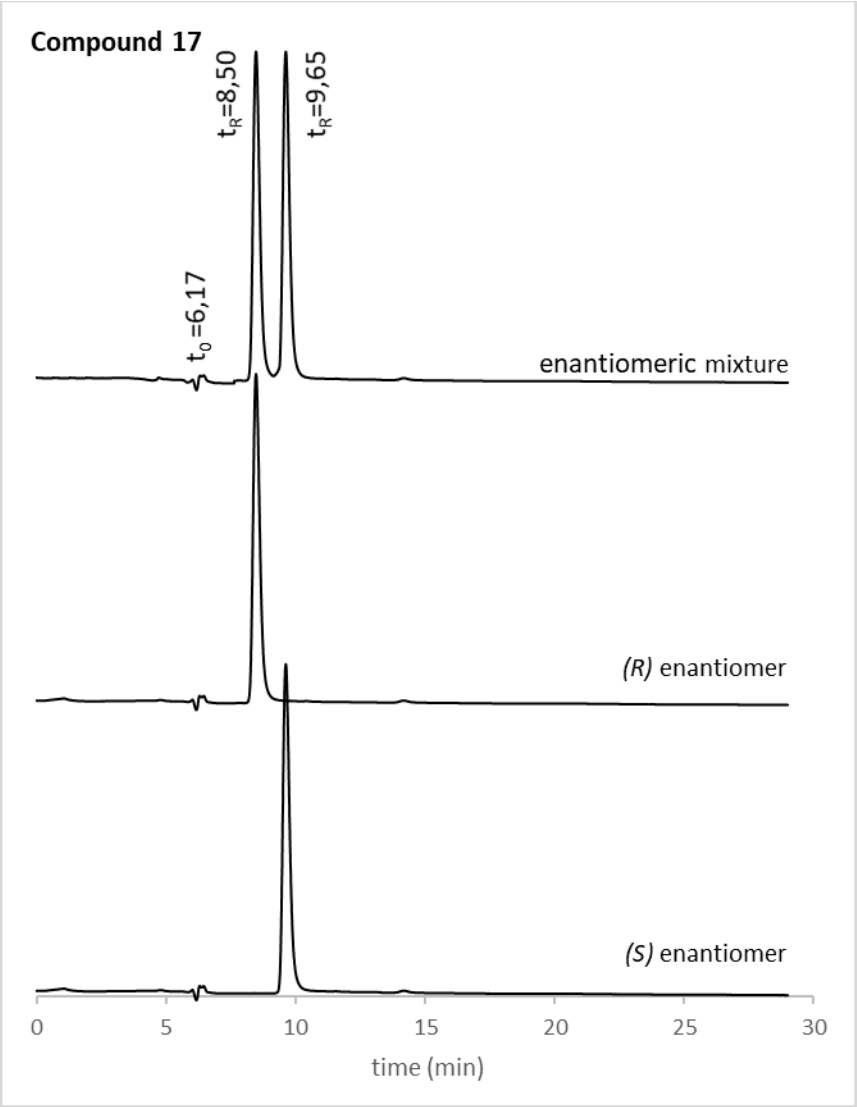

Figure S12. Chromatograms for the enantioseparation of the enantiomeric pair **17**, at optimized chromatographic conditions

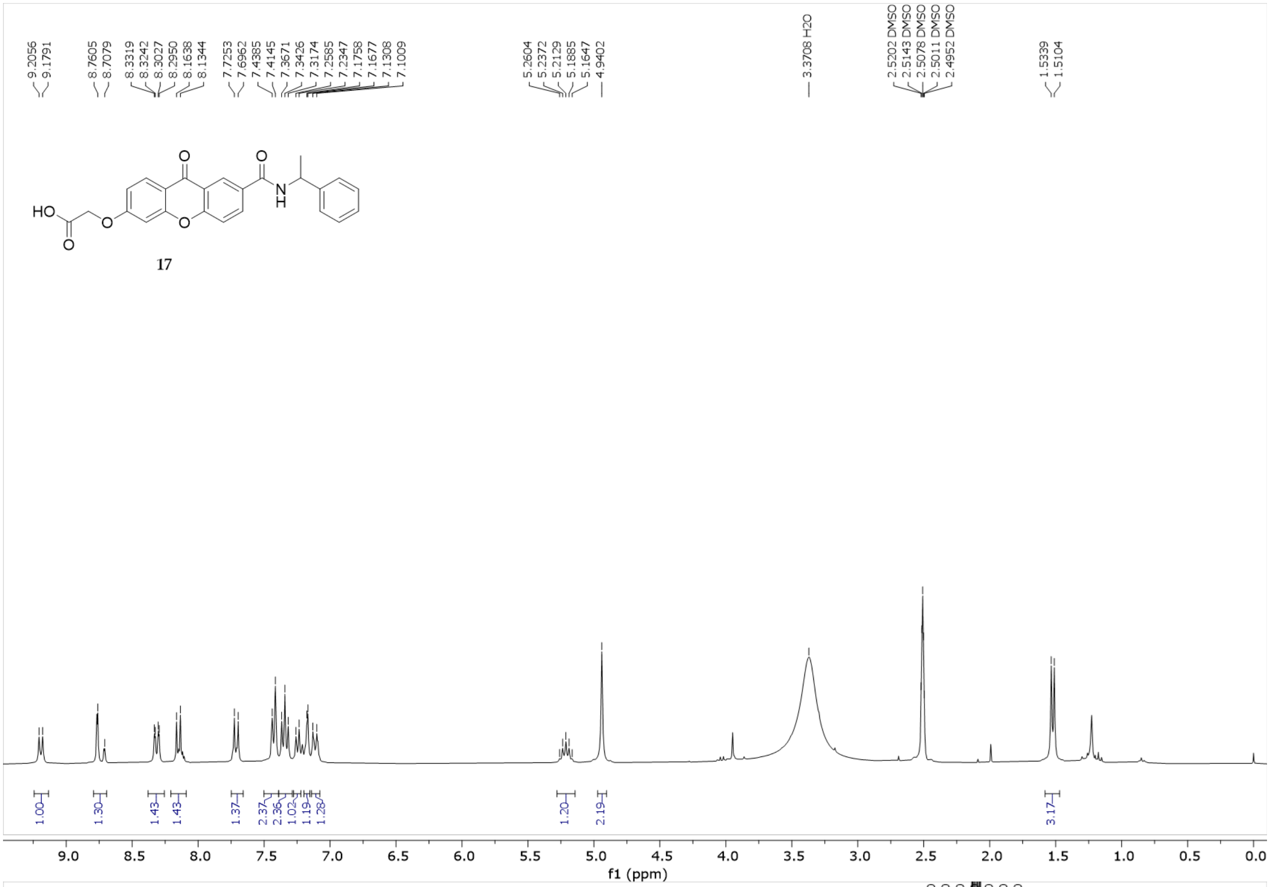

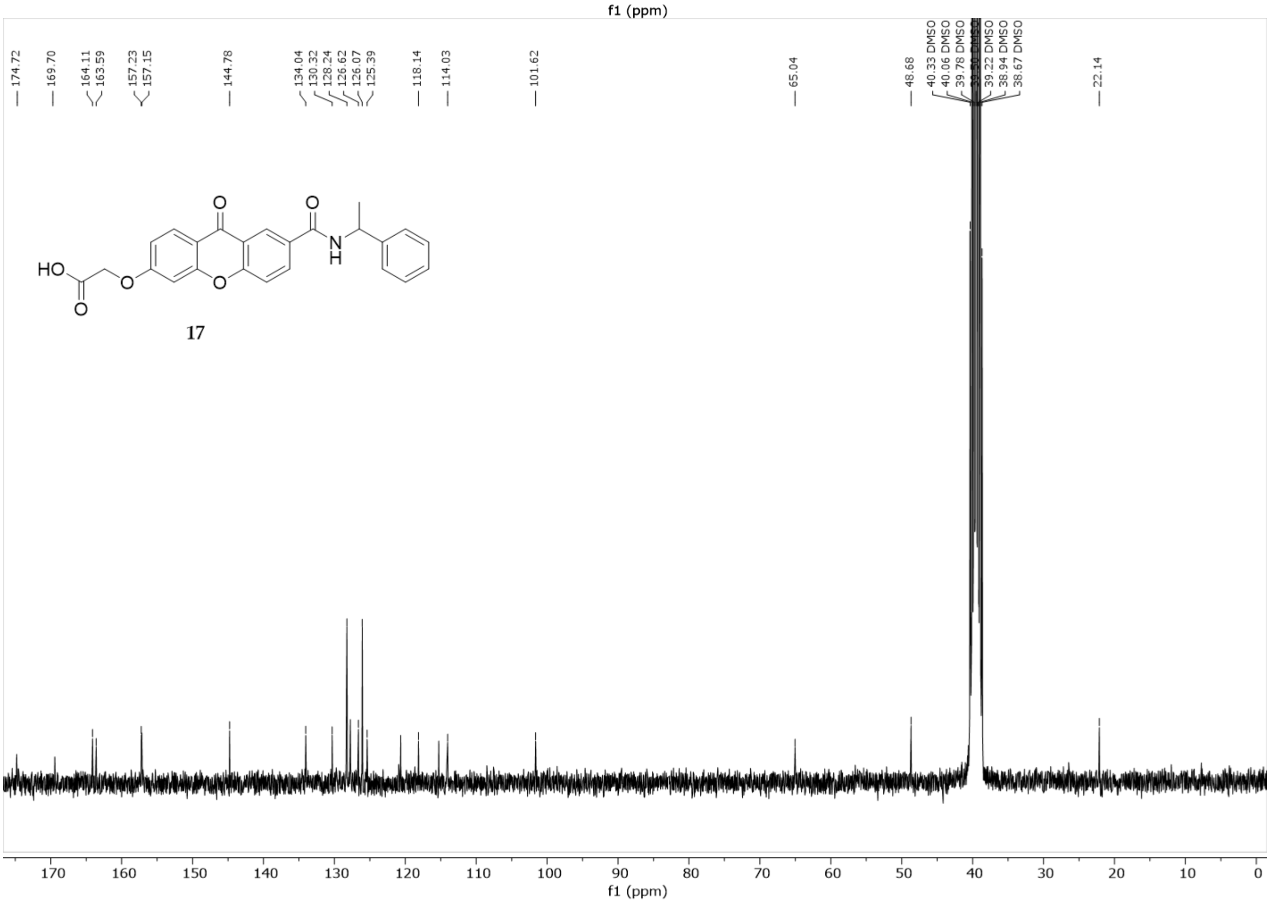

Figure S13. ^{1}H NMR (300.13 MHz, DMSO-*d*6) and ^{13}C NMR (75.48 MHz, DMSO-*d*6) for the enantiomeric pair **17**.

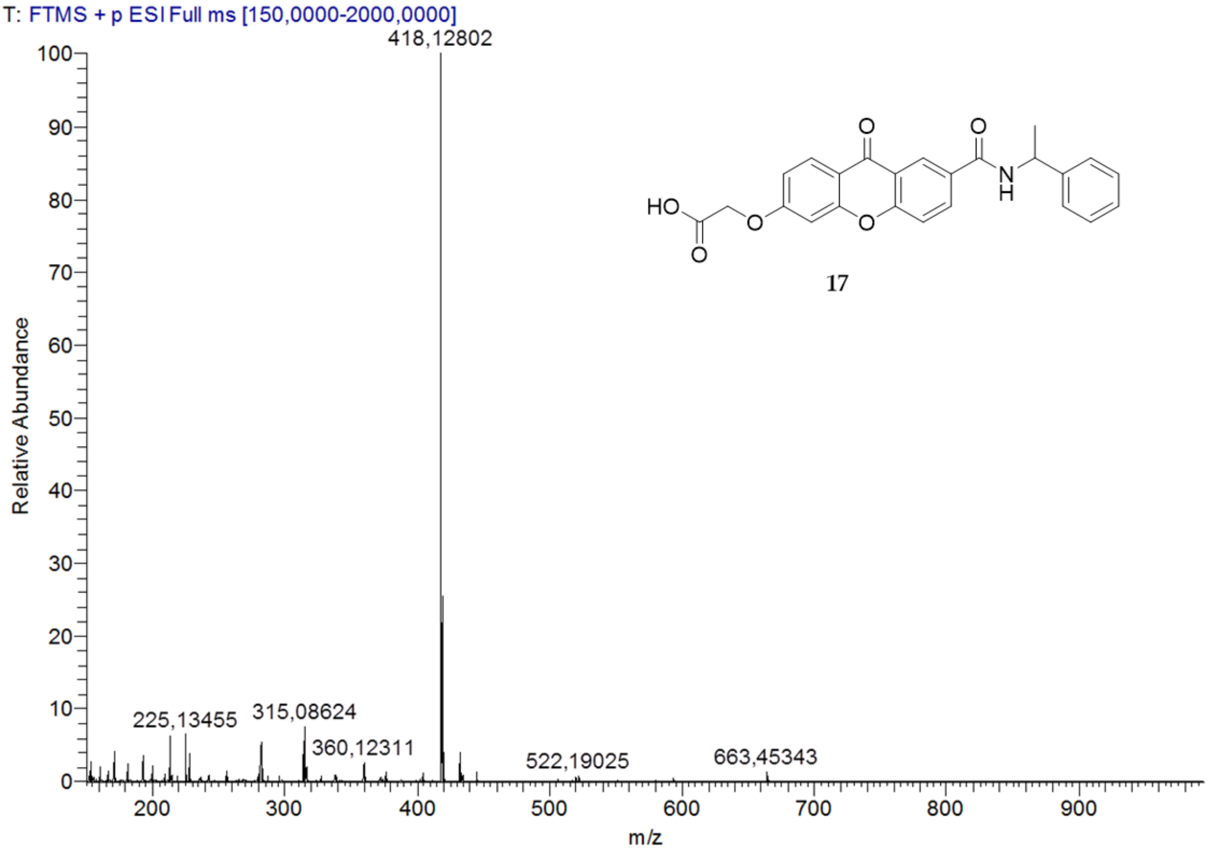

Meas. m/z	Formula	m/z	err [ppm]
418.12906	$C_{24}H_{20}NO_6$	418.12802	-1.191

Figure S14. Electrospray ESI data for the enantiomeric pair **17**.

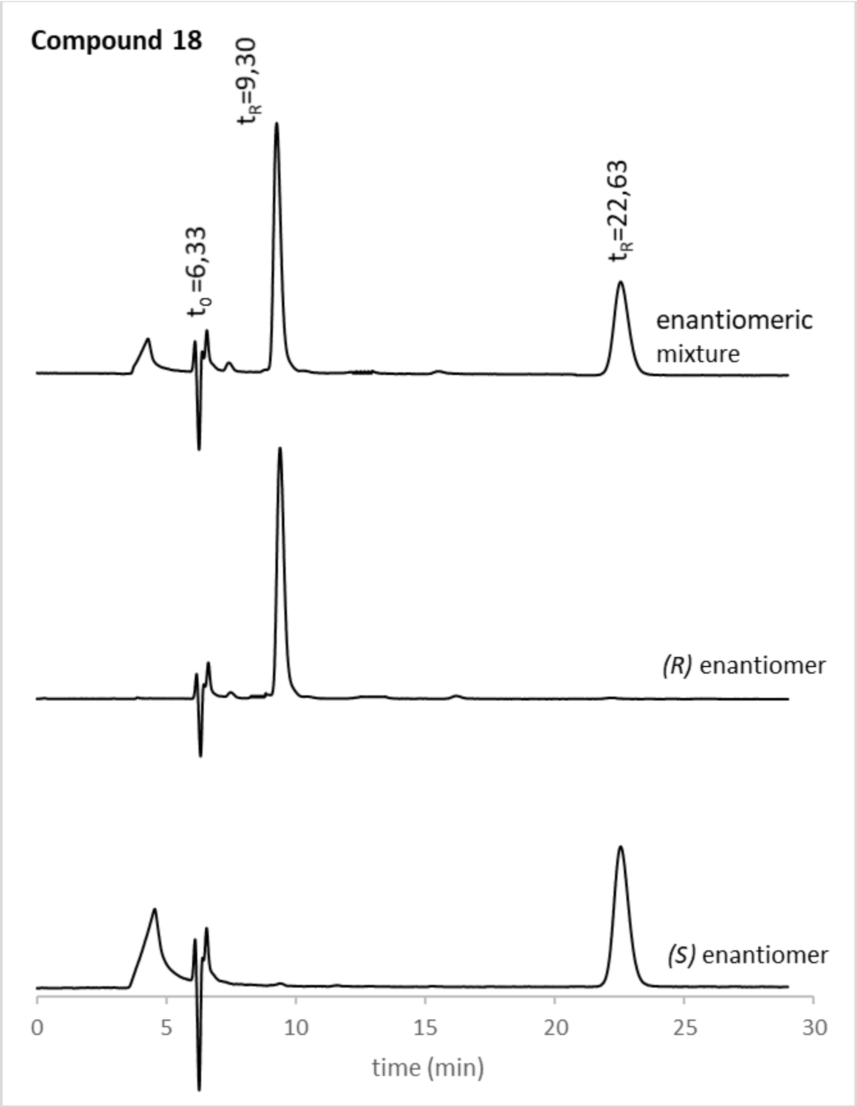

Figure S15. Chromatograms for the enantioseparation of the enantiomeric pair **18**, at optimized chromatographic conditions.

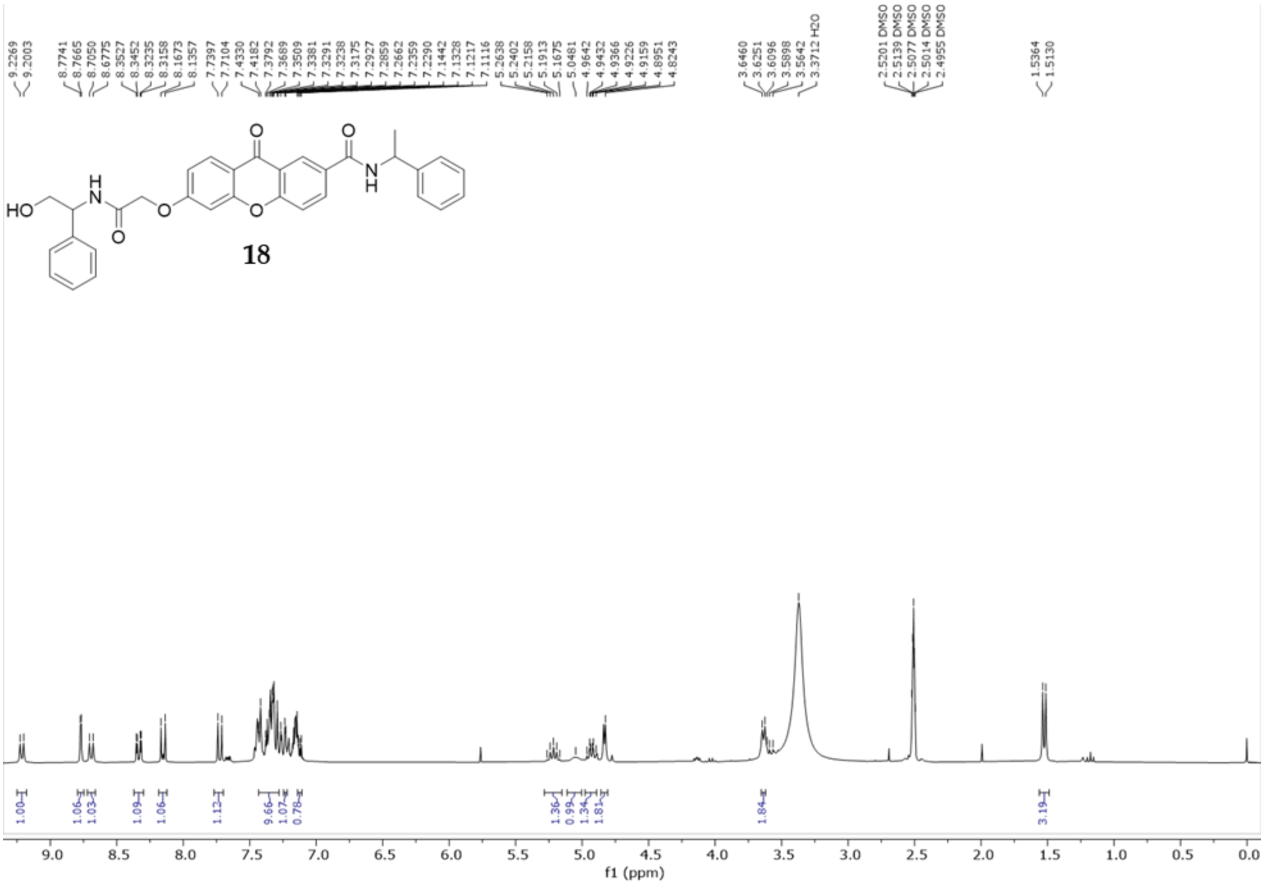

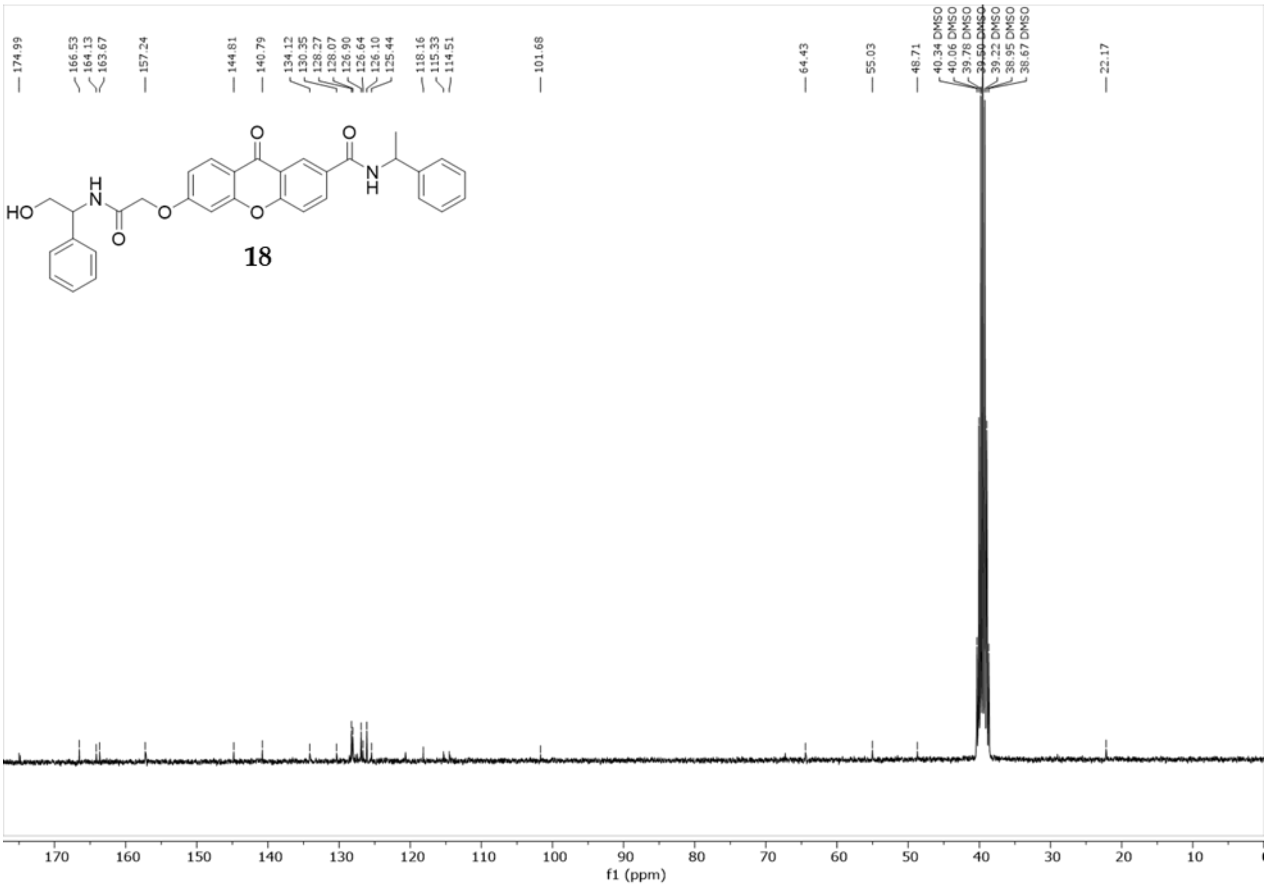

Figure S16. ^{1}H NMR (300.13 MHz, DMSO-*d*6) and ^{13}C NMR (75.48 MHz, DMSO-*d*6) for the enantiomeric pair **18**.

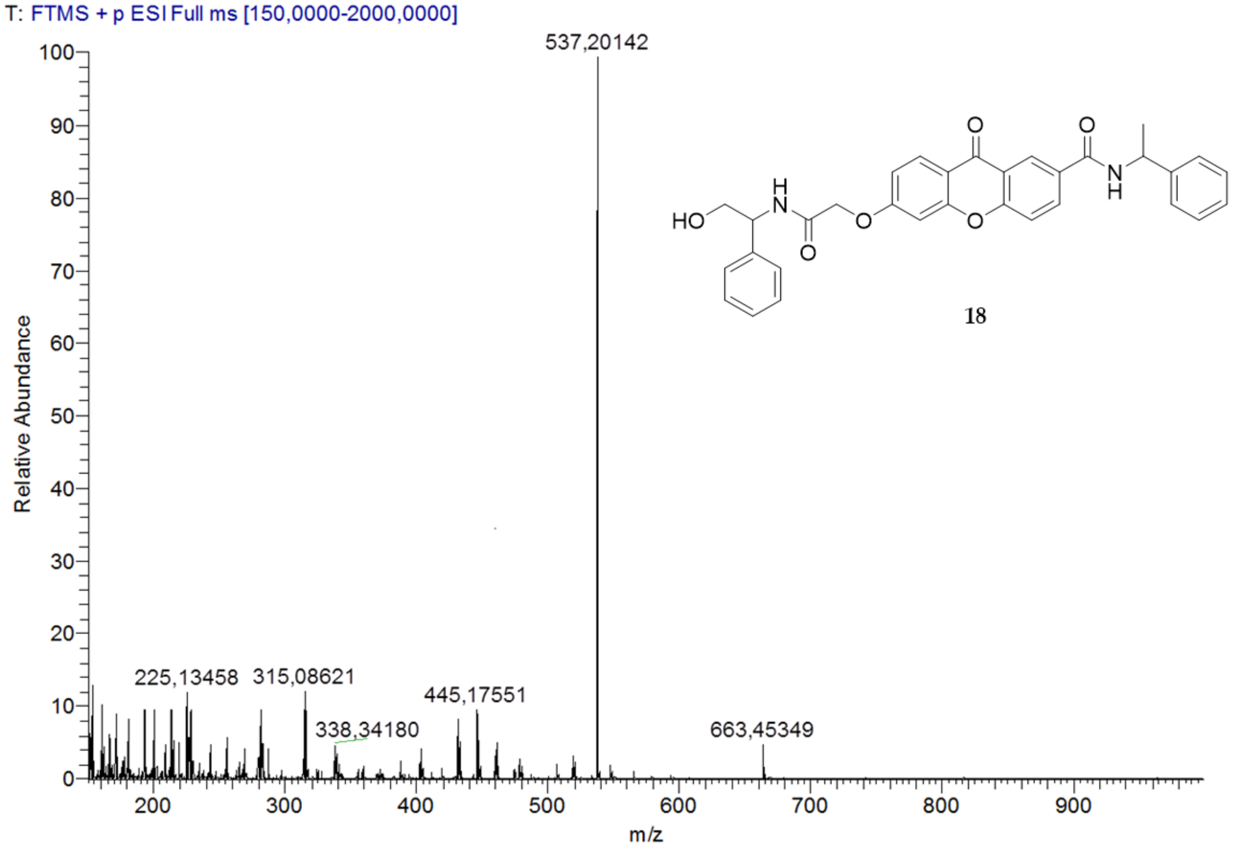

Meas. m/z	Formula	m/z	err [ppm]
537.20256	$C_{32}H_{29}N_2O_6$	537.20142	-1.104

Figure S17. Electrospray ESI data for the enantiomeric pair **18**.
